# Effects of molecular weight, end group of polylactide and coating thickness on corrosion rate and inhomogeneity of iron under biomimetic conditions

**DOI:** 10.1093/rb/rbag126

**Published:** 2026-06-13

**Authors:** Wenjie Wu, Jiandong Ding

**Affiliations:** State Key Laboratory of Molecular Engineering of Polymers, Department of Macromolecular Science, Fudan University, Shanghai 200438, China; State Key Laboratory of Molecular Engineering of Polymers, Department of Macromolecular Science, Fudan University, Shanghai 200438, China

**Keywords:** biodegradable stent, polymer coating, biometal corrosion, polylactide, Hank’s solution

## Abstract

Iron-based biodegradable stents hold significant promise for percutaneous coronary intervention. Our previous study has revealed that a polylactide (PLA) coating can not only accelerate iron corrosion but also mitigate corrosion inhomogeneity, presenting a metal-polymer composite stent (MPS). Nevertheless, current understanding of how PLA coating parameters regulate iron corrosion remains limited. This article represents the systematic and quantitative *in vitro* investigation into the effects of coating thickness, molecular weight, and end group of PLA on the corrosion rate and inhomogeneity of an iron substrate under biomimetic conditions. By theoretically developing an equation set to quantify random degradation with time-dependent inhomogeneity and experimentally combining immersion corrosion tests with electrochemical analysis, we elucidate the spatiotemporal evolution of iron corrosion under different coating parameters. Our findings provide a crucial foundation for optimizing the degradation behavior of MPS and stimulating the design of other biodegradable materials.

## Graphical Abstract

**Graphical Abstract:**
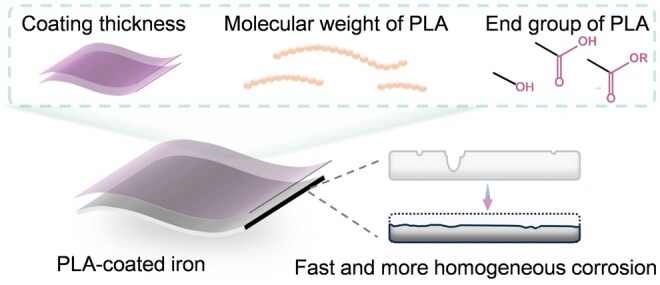

## Introduction

Percutaneous coronary intervention with biodegradable stents has been the mainstream treatment for coronary artery diseases owing to its minimal invasiveness [1–4]. An ideal biodegradable stent provides temporary mechanical support to the vessel after implantation, followed by gradual degradation and absorption by the body when the vessel undergoes repair and remodeling [5–8]. During degradation of the stent, vessels are reconstructed and physiological vasomotor function is restored [9–11]. Iron-based stents have garnered considerable attention with excellent mechanical properties [12–15]. Compared with polymeric materials and magnesium- or zinc-based substrates, the superior mechanical performance of iron facilitates the fabrication of stents with thinner struts [16–18], thereby reducing disturbance to blood flow [19–21]. The clinical translation of iron-based stents has long been hindered by two critical limitations: first, their excessively slow degradation rate under physiological conditions [22–25], which fails to align with the vascular remodeling; second, their susceptibility to localized corrosion, predominantly pitting corrosion [26, 27], which may lead to premature strut fracture, local protrusion, or mechanical discontinuity, ultimately precipitating adverse clinical events [28–31]. The thinner strut architecture renders iron stents more sensitive to corrosion inhomogeneity, where variations in local corrosion rates are more likely to create mechanically weak regions, further elevating clinical risks. These challenges have substantially impeded the full realization of iron’s potential as a next-generation stent material.

**Figure 1:**
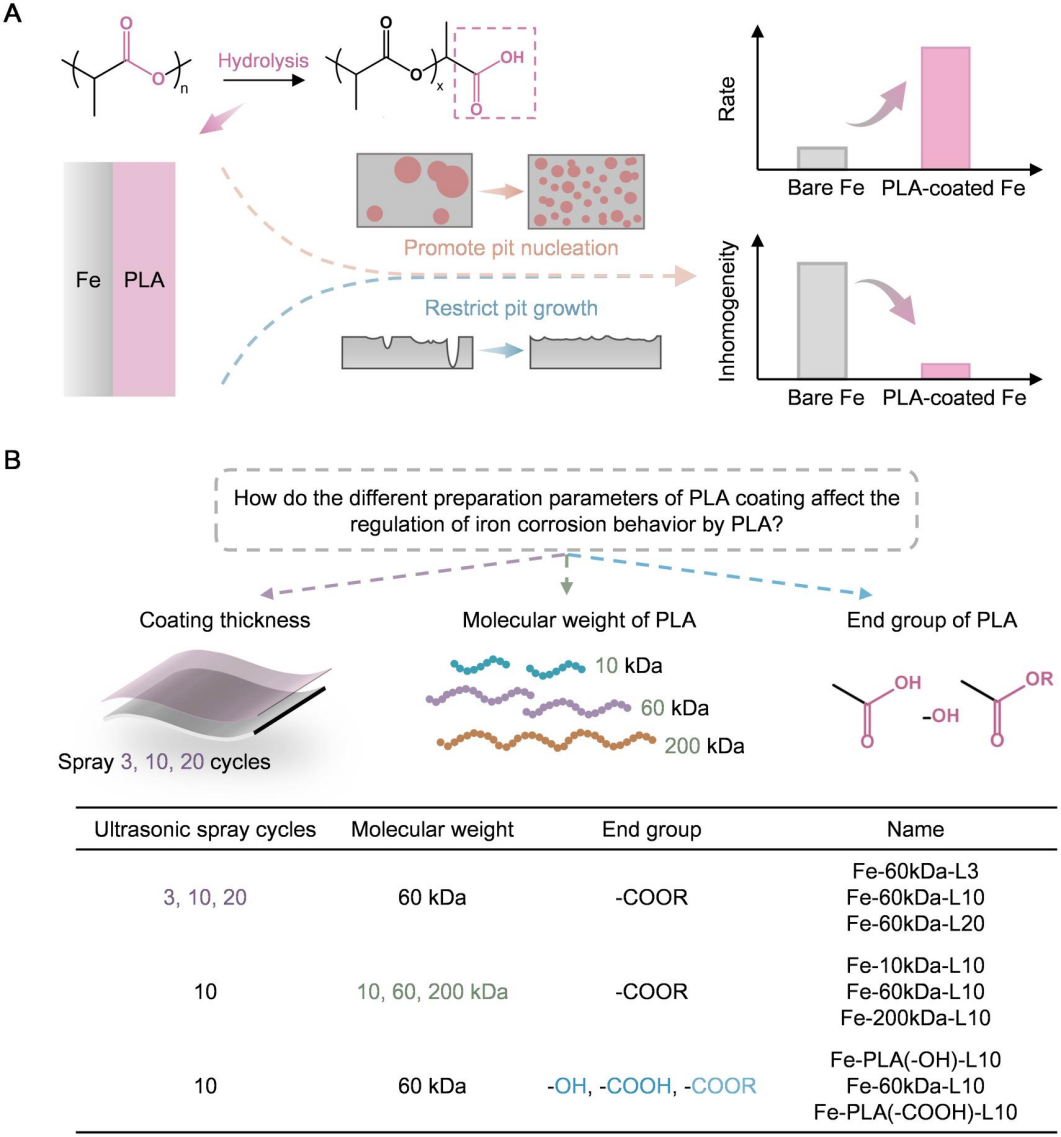
Significant modulation of iron corrosion by a PLA coating and the selection of coating parameters. (**A**) Schematic illustration of the corrosion behavior of iron regulated by the PLA coating. The polyester coating promotes the formation of corrosion pits while suppressing their excessive local longitudinal growth, thereby significantly increasing the corrosion rate of iron and reducing the corrosion inhomogeneity. (**B**) Effects of different coating preparation parameters on the corrosion regulation behavior of PLA-coated iron–selection of PLA molecular weight, PLA end group, and coating thickness as research parameters from the microscopic to the macroscopic level. As the shared experimental group, Fe-60kDa-L10 means Fe as substrate, 60 kDa MW of PLA, 10 layers during ultrasonic spraying.

Our early studies have demonstrated that a polylactide (PLA) coating offers a promising strategy to address these challenges [32–35], as schematically illustrated in Figure 1A. The PLA coating can significantly promote the otherwise overly slow corrosion by creating a localized acidic microenvironment with hydrolysis of the polyester [36–38]. In addition, preliminary qualitative observations suggest that the PLA coating may play a beneficial role in suppressing the longitudinal propagation of pits [39], implying that the metal-polymer composite stent (MPS) strategy holds the potential to simultaneously mitigate both excessive degradation rate and corrosion inhomogeneity. Currently, the globally first fully degradable iron-based coronary stent, IBS^®^, is at the stage of clinical trial [40, 41].

Although the modulating effect of PLA coatings on iron corrosion has been preliminarily validated, a systematic understanding of how coating parameters quantitatively influence the corrosion rate and inhomogeneity of the iron substrate has not been reported. Previous investigations of corrosion inhomogeneity are largely confined to qualitative descriptions [42–44]. In our prior work, we established a corrosion coverage—mass equation to describe corrosion inhomogeneity in metallic materials, and proposed the ‘half-corrosion mass’ (*M*_corro50%_) – the mass loss corresponding to 50% corrosion coverage–as a key parameter for quantitatively characterizing corrosion inhomogeneity [45]. A larger *M*_corro50%_ indicates that a greater mass loss is required to achieve the same corrosion coverage, reflecting a more spatially heterogeneous corrosion process. This theoretical framework represents a significant advancement in the quantitative study of corrosion inhomogeneity.

The present study further proposes the dynamic corrosion inhomogeneity, and quantitatively investigates the regulatory effects of PLA coating parameters on the corrosion rate and inhomogeneity of iron under biomimetic conditions. As depicted in Figure 1B, we varied coating thickness, molecular weight and end group of PLA across macroscopic to microscopic scales, and combined immersion corrosion tests with electrochemical analysis to elucidate the spatiotemporal evolution of the corrosion rate and inhomogeneity of iron under different parameter conditions. The plasma mimetic Hank’s solution serves as the corrosion medium in our *in vitro* study. Our findings provide critical theoretical insights for optimizing the degradation behavior of iron-based MPS, thereby laying a scientific foundation for advancing their clinical application. The new parameters might also be stimulating for quantifying time-dependent inhomogeneity of other systems with random degradation.

## Materials and methods

### The extended equation set of random degradation

**Figure 2:**
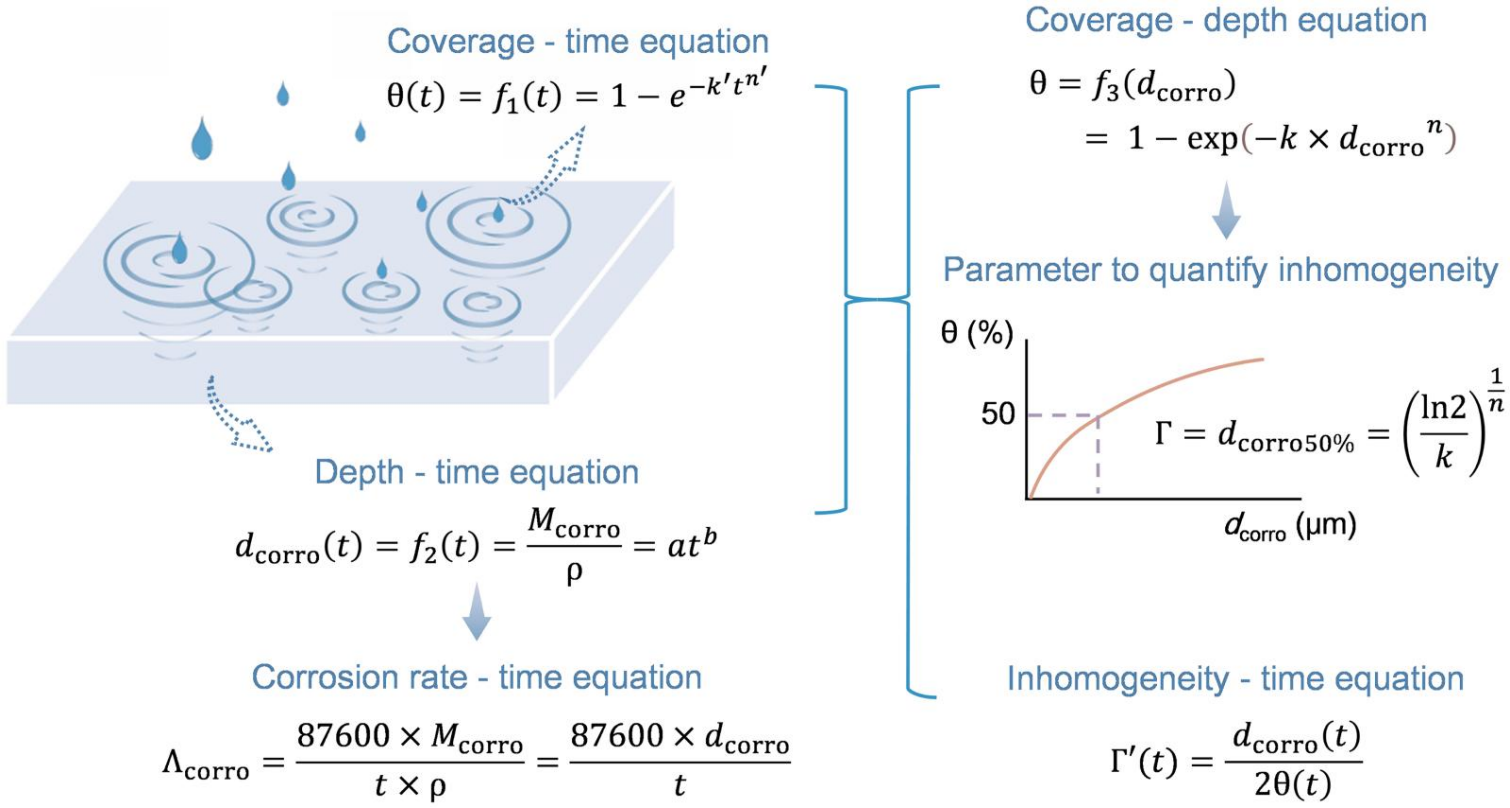
Schematic illustration of the equation set of random degradation. The quantitative description of corrosion rate and inhomogeneity is ingeniously established based on the Poisson raindrop model. First, the relationships between corrosion coverage (θ) and time (*t*), as well as corrosion depth (*d*_corro_) and *t*, are established separately. Then from the *d*_corro_-*t* equation, the corrosion rate Λ_corro_ at different *t* can be calculated. After combining the θ-*t* and *d*_corro_-*t* equations and eliminating *t*, the relationship between θ and *d*_corro_ is obtained. We define *d*_corro_ at 50% coverage as the static or default quantitative parameter Γ for evaluating corrosion inhomogeneity. Furthermore, the time-dependent or dynamic index Γ’(*t*) is introduced to track the temporal evolution of corrosion inhomogeneity.

Our equation set describing metal corrosion is illustrated in Figure 2. It is based on the theoretical framework proposed in our previous study [45, 46], which employs the Poisson raindrop model to characterize the corrosion process of metals. The relationships among the corrosion coverage (θ), corrosion depth (*d*_corro_) and corrosion time (*t*) are given as follows:


(1) θ(t)=f1(t)=1-e-k′tn′

(2)  dcorro(t)=f2(t)=atb

Here, θ refers to the ratio of the normal projected area of corrosion pits to the total projected area of the substrate surface, indicating the extent of corrosion on the substrate surface. *d*_corro_ is defined as the vertical distance from the original surface level, which is obtained by averaging over both corroded and uncorroded regions of the substrate. The vertical distance is ready to be calculated from the corrosion mass per unit area (*M*_corro_) over metal density (ρ).

Based on the determination of *d*_corro_ and referring to the standard ASTM G1-03, the corrosion rate (Λ_corro_) in units of mm/year is obtained as follows:


(3) Λcorro=87600×MA×t×ρ=87600×dcorrot

Here, *M* represents the total mass loss (g), *A* is the exposed surface area (cm^2^), *t* is the corrosion time (h), ρ is the metal density (g/cm^3^), and 87 600 is the unit conversion factor mainly from the length of year to that of hour.

By eliminating *t* from equations (1) and (2), the correlation between θ and *d*_corro_ is obtained:


(4) θ=f3(dcorro)=1-exp⁡(-k×dcorron)

In analogy with the definition of the half-crystallization time in the Avrami equation [47], *d*_corro_ corresponding to θ = 50% (*d*_corro50%_) is defined as a key parameter Γ, which quantifies corrosion inhomogeneity:


(5) $$\begin{array}{c} \Gamma =d_{\mathrm{corro}50\%}={\left(\frac{\ln2}{k}\right)}^{\frac{1}{n}} \end{array}$$

Furthermore, this article reports a dynamic parameter to describe the evolution of corrosion inhomogeneity over time:


(6) Γ'(t)=dcorro(t)θ(t)/50%

### Preparation of iron sheets and electrodes

High-purity iron sheets (99.99%) were progressively polished with sandpapers (SiC) of 800, 1500, 2000 and 3000 grit, ultrasonically cleaned in acetone and ethanol (10 min each), and dried under high-purity nitrogen gas. The sheets were then cut into 1.0 cm (length) × 1.0 cm (width) pieces and stored under vacuum.

For electrochemical experiments, the iron sheets were fabricated into working electrodes. A wire was soldered to the back of each sheet, and the rear side was encapsulated with epoxy resin inside a polyvinyl chloride (PVC) tube, leaving a fixed exposed area of 1.0 cm^2^. After resin curing, the exposed surface was polished with sandpapers of 800, 1500, 2000 and 3000 grit, ultrasonically cleaned again in acetone and ethanol, and dried with high‑purity nitrogen before use.

### Preparation of PLA coatings

PLA coatings were prepared via ultrasonic spraying of a 0.01 g/mL PLA solution in ethyl acetate at a syringe-pump feed rate of 0.05 mL/min. Solutions were prepared from PLA with ester end‑groups with molecular weight (MW) 10, 60 and 200 kDa, carboxyl end‑group (MW 60 kDa), and hydroxyl end‑group (MW 60 kDa) (Jinan Daigang Biomaterial Co., Ltd, China). The corresponding synthetic routes are presented in Supplementary Figure S1. D, L-lactide was fed prior to ring opening polymerization. As a result, while one end groups were kept hydroxyl, the other end groups differed, which was controlled by using different initiators.

The molecular weights of all types of PLA were characterized by gel permeation chromatography (GPC), and their end-group structures were confirmed by nuclear magnetic resonance (NMR) spectroscopy. To examine the effect of coating thickness, the 60 kDa ester‑terminated PLA was sprayed with 3, 10 and 20 cycles. All coated samples were dried overnight in a fume hood to allow complete solvent evaporation.

### Characterization of PLA coatings

Surface wettability of bare iron and PLA-coated samples was assessed via contact angle measurements at three random locations per group. The chemical composition of the PLA coatings was analyzed by Fourier transform infrared spectroscopy (FTIR) using an attenuated total reflection (ATR) accessory. Spectra were acquired from 4000 to 500 cm^−1^ for both coated and uncoated surfaces.

Surface morphology was examined using a lanthanum hexaboride scanning electron microscope (LaB_6_-SEM) operated at 15 kV in secondary electron (SE) mode to minimize beam-induced damage to the polymer.

Coating thickness was determined with a step profiler by scanning perpendicular to the coating-substrate interface and measuring the height difference between coated and exposed substrate regions. Prior to measurement, the specimens were prepared as follows: half of each iron sheet was masked with A4 paper during spraying, leaving one half coated and the other half as bare metal.

### Influence of different coating parameters on the corrosion rate of iron

The backside of sheet samples was sealed with silicone rubber to confine exposure to the coated or control surface. Samples were immersed in Hank’s solution, which was employed as the simulated physiological fluid, with the following composition and concentrations: sodium chloride (8.00 g/L), glucose (1.00 g/L), magnesium sulfate (0.10 g/L), magnesium chloride (0.10 g/L), disodium hydrogen phosphate dodecahydrate (0.12 g/L), potassium dihydrogen phosphate (0.06 g/L), sodium bicarbonate (0.35 g/L), potassium chloride (0.40 g/L), and calcium chloride (0.14 g/L). After preparation, the solution pH was adjusted to 7.40 ± 0.05. Each group (*n *= 3) was incubated in 10 mL solution within a shaking incubator (37 °C, 50 rpm), with solution replacement every two days. For electrode samples, 500 mL of Hank’s solution was used as the immersion medium.

Corrosion rate was obtained through corrosion mass loss experiments and calculated according to equation (3). After 4 h, 1 d, 3 d, 7 d, 15 d and 30 d of immersion, the corrosion mass loss was determined from the ion concentration measured with inductively coupled plasma-optical emission spectrometry (ICP-OES). The Hank’s solution, which was replaced every two days, was collected and retained for the determination of soluble corrosion products. Insoluble corrosion products were dissolved according to GB/T 16545‑2015 using a solution containing 3.5 g/L hexamethylenetetramine in hydrochloric acid and collected in centrifuge tubes. For ICP-OES analysis, samples were acidified with dilute nitric acid, diluted to a fixed volume, and filtered through a 0.22 μm membrane. A calibration curve was generated from iron standard solutions (10, 20, 50, 100 and 200 ppm). Iron concentration was determined based on characteristic spectral line intensity. Corrosion mass per specimen was calculated from the measured ion concentration and solution volume. Background correction was applied using unimmersed controls subjected to the same cleaning procedure.

Polarization curves were obtained at 0.5 h, 4 h, 1 d, 3 d and 7 d in a three‑electrode cell with saturated calomel electrode and Pt counter electrode. Before each test, specimens were stabilized at open circuit potential (OCP) for 10 min. Scans were performed from −400 mV to +400 mV vs OCP at 0.33 mV/s in Hank’s solution at 37 °C. Corrosion current densities were derived by Tafel extrapolation.

### Measurement of corrosion coverage

Optical morphology was examined before immersion and after 4 h, 1 d, 3 d, 7 d, 15 d and 30 d of immersion. Corrosion morphology and coverage on iron sheets after 15 d were further characterized with LaB_6_-SEM. Prior to scanning electron microscopy (SEM) observation, corrosion products were chemically removed using a solution containing 3.5 g/L hexamethylenetetramine in hydrochloric acid, followed by rinsing and drying at room temperature. Corrosion coverage was quantitatively evaluated from optical images using ImageJ software at each time point.

### Measurement of corrosion depth

Corrosion depth was derived from iron ion concentration measured by ICP-OES. Given a fixed corrosion area of 1.0 cm^2^, the results represent corrosion mass per unit area, which was then converted to corrosion depth using the density of iron (7.86 g/cm^3^).

### Influence of different coating parameters on the corrosion inhomogeneity of iron

After 15 days of immersion in Hank’s solution, the cross-sectional morphology of each sample group was characterized with LaB_6_-SEM to analyze corrosion depth and corrosion inhomogeneity. The samples were prepared by sectioning perpendicular to the corroded surface and polishing with SiC sandpaper to obtain a flat observation plane. The corrosion coverage and corrosion depth data obtained at different immersion times were fitted using equation (4). The corrosion depth corresponding to 50% coverage, denoted as *d*_corro50%_, was derived from the fitted curve as a quantitative parameter Γ for corrosion inhomogeneity.

### Exploration of mechanism of PLA coating in regulating iron corrosion

A PLA coating (0.1 g/mL in ethyl acetate) was cast onto pretreated iron substrates and dried in air. After 3 d immersion in Hank’s solution, the coating was peeled off for interfacial analysis. The pH at the metal‑coating interface was measured with pH pencils and determined by colorimetric comparison.

In parallel, elemental composition at the interface was examined via energy dispersive spectroscopy (EDS) coupled with LaB_6_-SEM, focusing on uncorroded regions.

### Evolution of the corrosion process during full immersion

Electrochemical impedance spectroscopy (EIS) was measured under identical conditions at 0 min, 4 h, 1 d and 7 d, over a frequency range of 100 kHz to 0.01 Hz with 20 mV amplitude. Data were fitted to equivalent circuits using ZSimpWin software to extract component values.

### Statistical analysis

Statistical differences among sample groups were analyzed using one‑way analysis of variance (One-way ANOVA). The significance levels are indicated as follows: ‘***’ for *P* < 0.001, ‘**’ for 0.001 ≤ *P* < 0.01, ‘*’ for 0.01 ≤ *P* < 0.05 and ‘ns’ for *P* ≥ 0.05.

## Results

### Threefold corrosion acceleration of iron by varying PLA coating parameters

Equations (1–6) describe degradation rate, static and dynamic degradation inhomogeneity during random degradation. Our *in vitro* corrosion study justifies these fundamental regulations. All types of PLA used in this study were custom-ordered, with different terminal groups introduced via the use of distinct initiators. The measured molecular weights along with structural characterizations are shown in Supplementary Figure S2. The coating preparation parameters and the corresponding sample nomenclature are presented in Figure 1B. The coating thickness was controlled by adjusting the number of ultrasonic spraying cycles, which were set at 3, 10 and 20 cycles.

**Figure 3:**
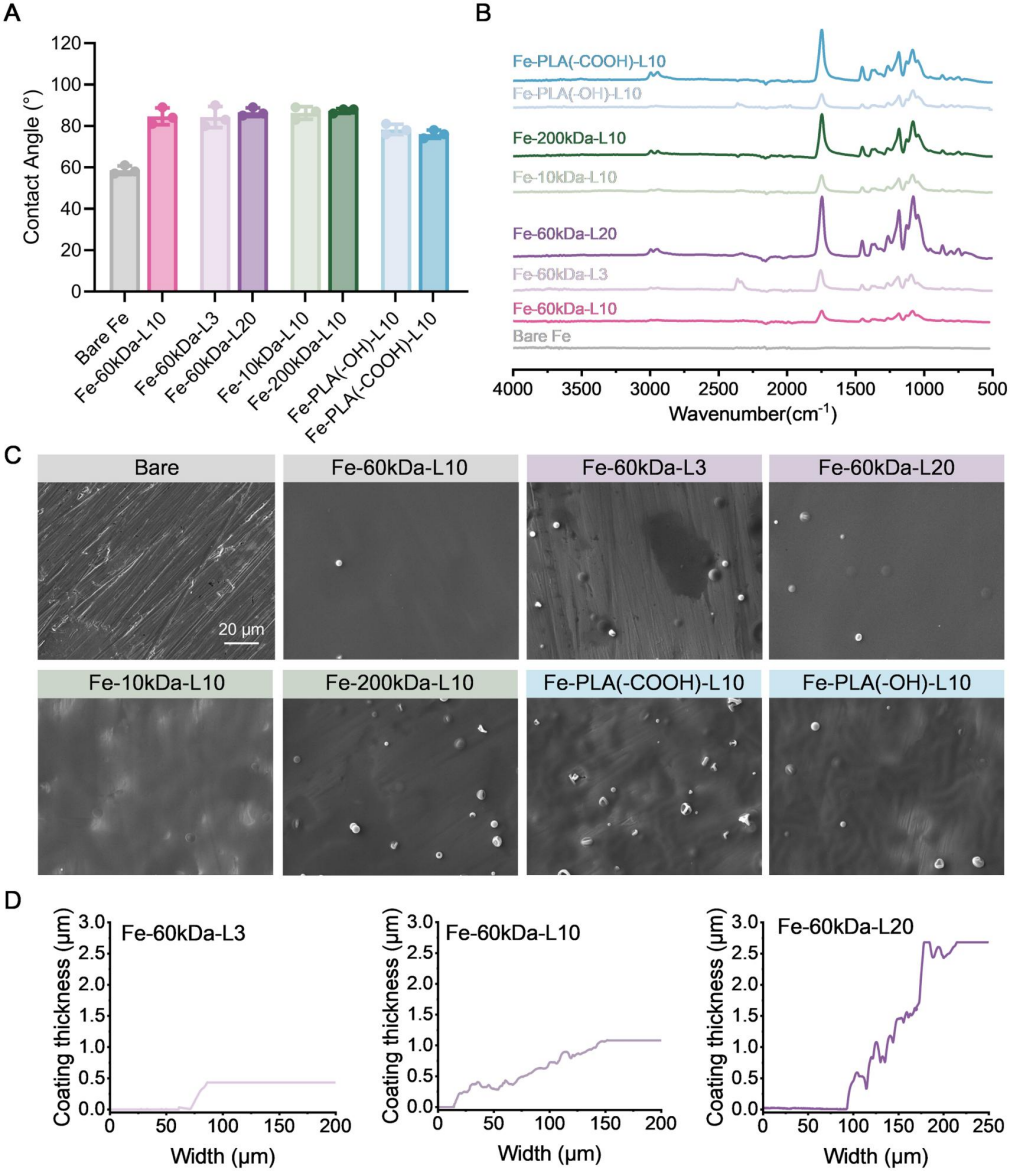
Preparation of PLA coatings. (**A**) Contact angles of PLA-coated iron with different coating parameters. (**B**) FT-IR spectra of PLA coatings with different variables. (**C**) SEM images of PLA coatings with different variables. (**D**) Thickness of PLA coatings with different ultrasonic spray cycles.

To verify the successful coating of PLA, a systematic physicochemical characterization was conducted on the coating samples prepared under various parameters. Contact angle measurements (Figure 3A) indicated that the PLA-coated iron surfaces exhibited significantly enhanced hydrophobicity, with the resultant contact angles markedly higher than those of bare iron. FTIR analysis (Figure 3B) revealed distinct characteristic absorption peaks on all PLA‑coated surfaces, whereas almost no infrared absorption signals were detected on the bare iron. SEM observation (Figure 3C) demonstrated that the bare iron surface displayed clear mechanical grinding marks, while the PLA-coated samples exhibited generally smooth and uniform surfaces with only a few solvent‑evaporation‑induced bubbles. Moreover, changes in either molecular weight or end group of PLA could alter the viscosity of the spraying solution, thereby affecting the resulting coating morphology. Thickness measurements of coatings obtained with different spraying cycles (Figure 3D) showed that 3, 10 and 20 spray cycles yielded coating thicknesses of approximately 0.4 μm, 1.1 μm and 2.6 μm, respectively.

**Figure 4:**
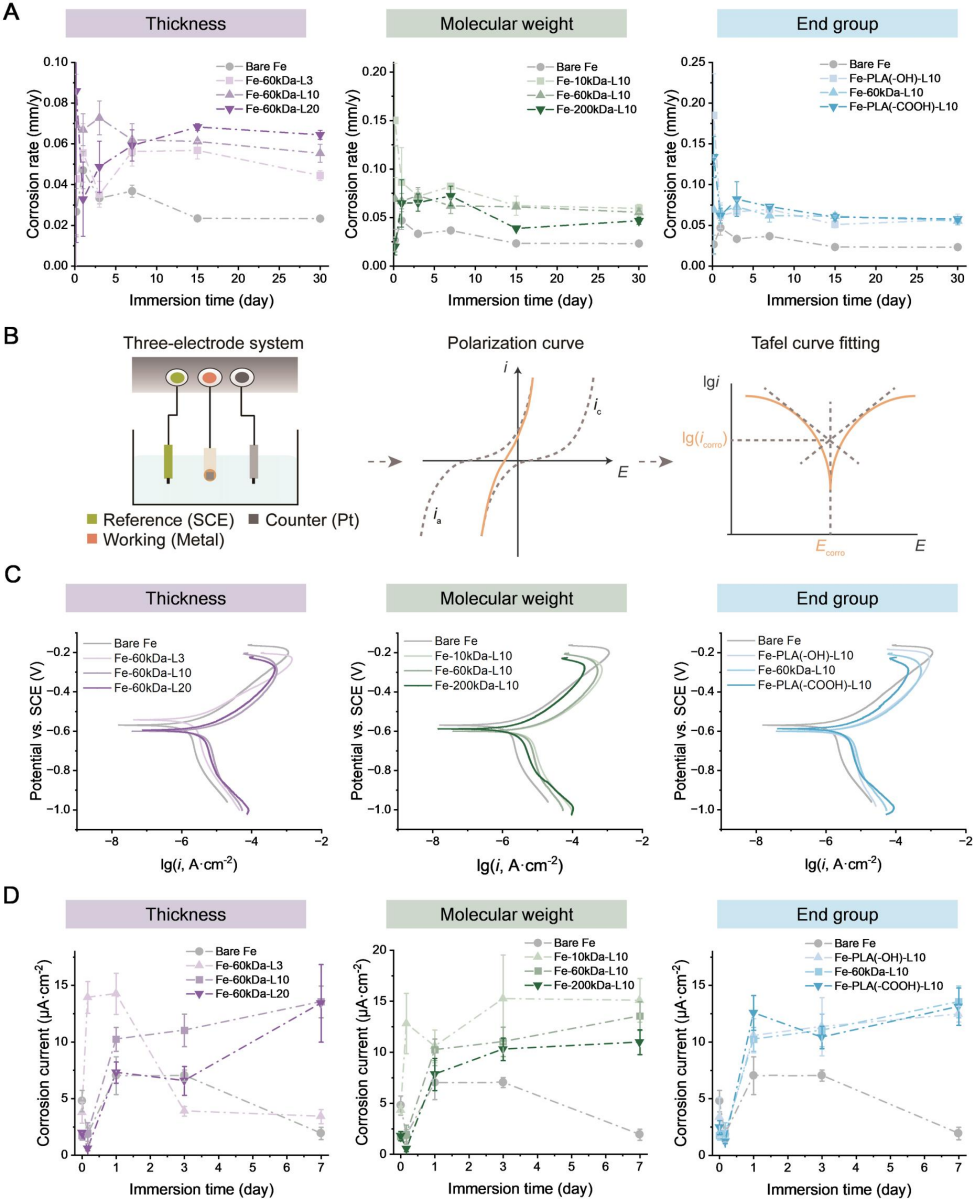
Corrosion rate of PLA-coated iron sheets with different parameters over immersion time in Hank’s solution. (**A**) Corrosion rates of different sample groups over immersion time, as determined by mass loss measurements. (**B**) Schematic diagrams of the three-electrode system and the Tafel curve fitting method for corrosion current density determination. Here, *i*_a_ and *i*_c_ represent the anodic and cathodic current densities, respectively, and *E*_corro_ denotes the corrosion potential. (**C**) Tafel curves of different sample groups after immersion in Hank’s solution for 7 days. (**D**) Corrosion current densities for all sample groups after varying immersion times in Hank’s solution.

The corrosion rates, calculated from time dependence of mass loss with equation (3), are presented in Figure 4A. After 30 days of immersion in Hank’s solution, all PLA-coated groups exhibited corrosion rates 2–3 times higher than the bare iron, confirming that the PLA coating significantly accelerates iron corrosion. Corrosion rate generally increased with greater thickness. Notably, the thickest coating (Fe-60kDa-L20) showed a slightly lower corrosion rate during the initial 7 days due to the physical barrier effect. After 7 days, the corrosion rate gradually exceeded those of other groups and remained the highest thereafter.

Lower molecular weight of PLA exhibited a more pronounced accelerating effect on corrosion, though this effect diminished with prolonged immersion time. Regarding end group, samples with carboxyl- and hydroxyl-terminated PLA showed slightly higher corrosion rates than those with ester-terminated PLA.

Electrochemical measurements were further employed to evaluate the corrosion rate. As shown in Figure 4B, polarization curves were obtained using a standard three-electrode system, and the corrosion current (*i*_corro_) was calculated by Tafel extrapolation, which is proportional to the corrosion rate [48, 49]. The Tafel curves for all samples after being immersed in Hank’s solution for 0.5 h, 4 h, 1 d, 3 d and 7 d are presented in Supplementary Figures S3–S5 and Figure 4C, with the corresponding *i*_corro_ values summarized in Figure 4D. In the initial stage, all PLA-coated samples exhibited a transient corrosion inhibition effect on the iron electrode, with *i*_corro_ lower than that of the bare iron.

Regarding coating thickness, after 4 h of immersion, the thinnest coating (Fe-60kDa-L3) was the first to transition to corrosion acceleration, though its accelerating effect gradually diminished thereafter. The thickest coating (Fe-60kDa-L20) exhibited the strongest corrosion promotion at 7 d. Regarding molecular weight, at 4 h of immersion, the Fe-60kDa-L10 and Fe-200kDa-L10 still showed inhibition, while the Fe-10kDa-L10 had already transitioned to acceleration and maintained the highest corrosion current throughout the entire immersion period. Regarding end group, all samples exhibited similar corrosion currents and potentials at each time point, transitioning from inhibition to acceleration after 1 d of immersion. Overall, all PLA coatings increased the *i*_corro_ of iron to some extent.

### Three-order-of-magnitude suppression of corrosion inhomogeneity in iron by varying PLA coating parameters

The global views of different samples before immersion and after immersion in Hank’s solution for 4 h, 1 d, 3 d, 7 d, 15 d and 30 d are presented in Supplementary Figure S6. All of the PLA coatings significantly increased the corrosion coverage of iron. At the same immersion time point, thicker coatings or lower molecular weight of PLA resulted in higher corrosion coverage. The surface morphologies after removal of corrosion products at 15 d of immersion are shown in Supplementary Figure S7. Compared with the bare iron surface, which exhibited only sporadic corrosion pits, thicker PLA coatings led to a higher number and denser distribution of corrosion pits on the sample surface. Furthermore, samples of higher molecular weight of PLA showed fewer corrosion pits, with some areas still exhibiting the original polishing marks. No obvious difference was observed among groups with different end group.

**Figure 5:**
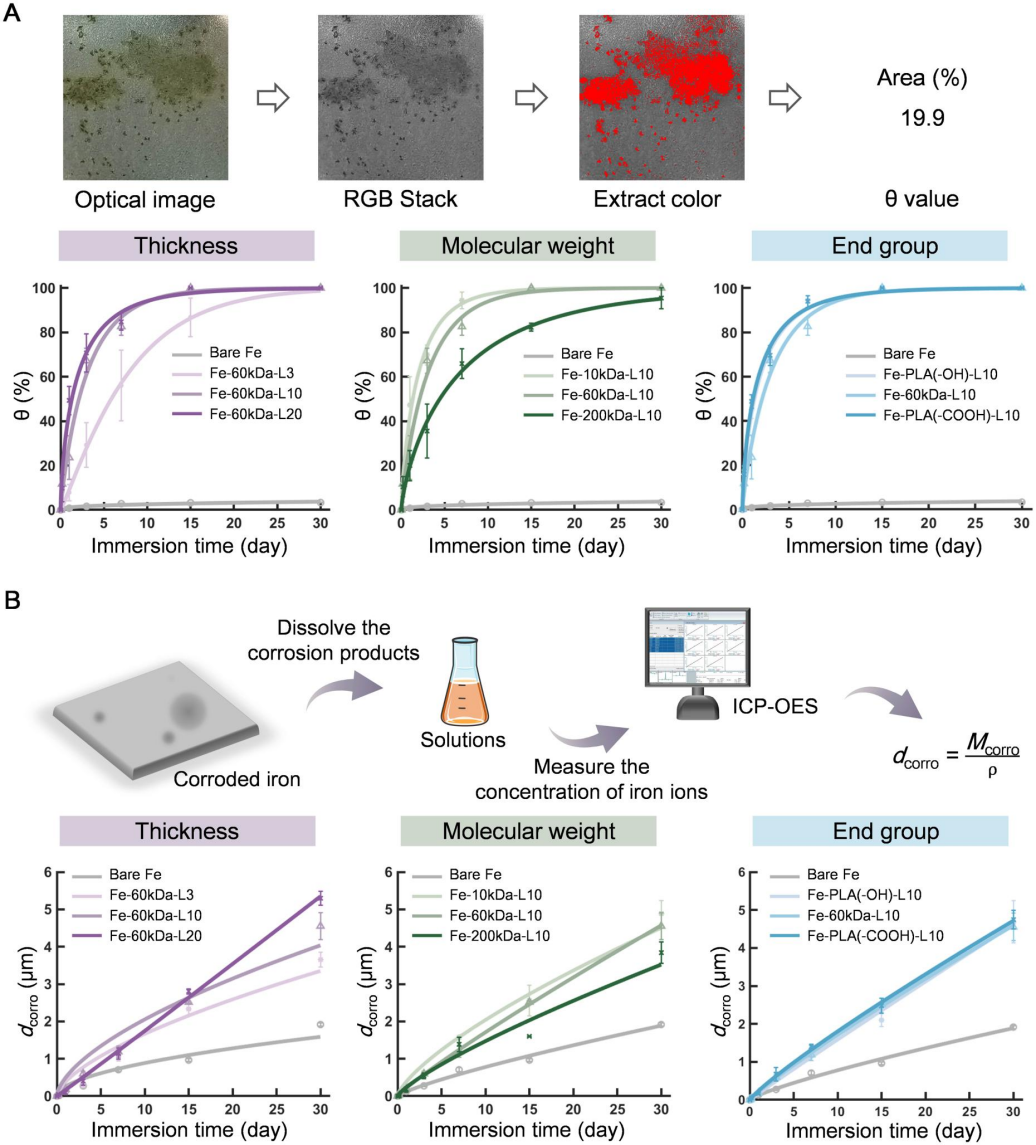
Corrosion coverage and depth versus time and the fitted curves with corresponding equations. (**A**) Schematic diagram of the corrosion coverage calculation process (top row) and corrosion coverage of PLA-coated iron sheets with different parameters, fitted using equation (1) (bottom row). (**B**) Schematic diagram of the corrosion depth calculation process (top row) and corrosion depth of PLA-coated iron sheets with different coating parameters in Hank’s solution, and the fitted curves by equation (2) (bottom row). All samples were immersed for 30 days in Hank’s solution.

Based on the optical images, the corrosion coverage was statistically analyzed and fitted using equation (1), with the results presented in Figure 5A. Greater thickness led to a more pronounced promotion of corrosion coverage. Lower molecular weight resulted in a more significant increase in corrosion coverage. The hydroxyl- and carboxyl-terminated groups exhibited similar promoting effects, both slightly superior to that of the ester-terminated group.

The fitted curves of corrosion depth for samples with different coating parameters using equation (2) are presented in Figure 5B, reflecting the influence of coating parameters on the longitudinal propagation of iron corrosion. Thicker coatings initially delayed corrosion propagation due to physical barrier effects. With prolonged immersion, their higher PLA content led to a sustained acidic microenvironment from degradation, resulting in the most significant corrosion promotion. Lower molecular weight coatings exhibited a stronger promoting effect on corrosion depth. No significant difference in corrosion depth was observed among different functional end groups.

Cross-sectional SEM observations were conducted on samples after 15 d of immersion in Hank’s solution to qualitatively assess corrosion inhomogeneity, with results shown in Supplementary Figure S8. Compared with the bare iron, which exhibited remarkable pitting corrosion, all PLA coatings markedly improved the flatness of the corrosion front. Regarding coating thickness, Fe-60kDa-L20 exhibited the flattest corrosion front, indicating optimal corrosion homogeneity, while Fe-60kDa-L3 still showed slight undulations. Regarding molecular weight, Fe-10kDa-L10 displayed the highest overall flatness of the corrosion front. Regarding end group of PLA, all three end groups exhibited similar flatness of the corrosion front.

**Figure 6:**
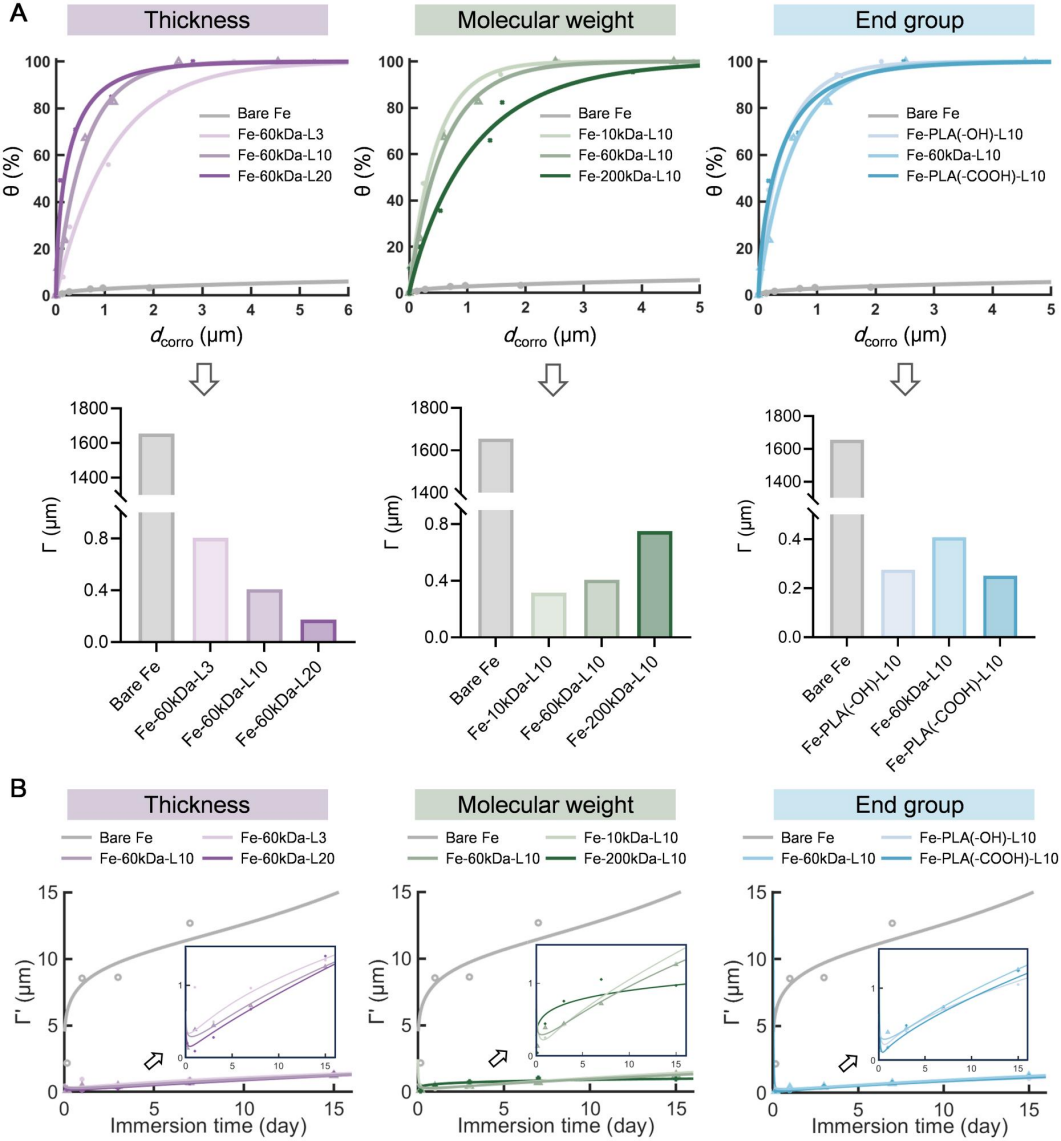
Corrosion inhomogeneity of PLA-coated iron sheets in Hank’s solution. (**A**) Corrosion coverage versus corrosion depth curves for PLA-coated iron sheets with different parameters (top row) and the corresponding Γ values for each substrate (bottom row). All samples were immersed for 30 days in Hank’s solution. **(B)** Time evolution of the dynamic corrosion inhomogeneity index Γ’(*t*). Smaller Γ’(*t*) indicates more uniform corrosion.

Subsequently, a quantitative method was employed to compare the corrosion inhomogeneity. The corrosion coverage—depth curves were fitted using equation (4), from which the quantitative parameter Γ at 50% corrosion coverage was calculated with results shown in Figure 6A. A lower Γ indicates less corrosion inhomogeneity and a more uniform corrosion process. PLA coatings reduced the corrosion inhomogeneity of iron by more than three orders of magnitude. Thicker coatings resulted in better corrosion homogeneity, with the Γ of the Fe-60kDa-L20 decreasing by more than 9000-fold compared with bare iron, demonstrating the most significant homogenization effect. Low molecular weight groups exhibited lower corrosion inhomogeneity, likely due to their faster degradation, which released more H^+^ during the experimental period, promoting surface pit initiation and inhibiting longitudinal pit growth. Hydroxyl- and carboxyl-terminated PLA showed better homogenization effects compared with ester-terminated PLA, with carboxyl-terminated slightly outperforming hydroxyl-terminated. This difference may be attributed to the influence of end-group polarity, hydrophilicity, and autocatalytic ability on PLA hydrolysis rate [50].

To dynamically track the evolution of corrosion inhomogeneity over time, we define a dynamic corrosion inhomogeneity index Γ’(*t*) as expressed in equation (6). Unlike the static parameter Γ, Γ’(*t*) reflects the average corrosion depth per unit coverage in real time, revealing the temporal evolution of corrosion’s spatial distribution. A smaller Γ’(*t*) indicates more uniform corrosion distribution, while a higher value signifies greater inhomogeneity. Figure 6B shows the Γ’(*t*) curves of PLA-coated iron with different parameters as a function of immersion time. Overall, all PLA-coated groups exhibited substantially lower Γ’(*t*) compared with bare iron, indicating that PLA coating fundamentally improves corrosion inhomogeneity. The evolution trends differed markedly. Γ’(*t*) of bare iron increased from approximately 2 μm initially to 15 μm at 15 d, an approximately 7-fold increase. In contrast, the values in the PLA-coated groups remained consistently at or below approximately 1 μm. During the 15 d immersion, thicker coatings maintained lower Γ’(*t*), indicating a more significant inhibition effect. Over time, Γ’(*t*) of the Fe-60kDa-L20 group showed a trend of approaching those of thinner coating groups, suggesting a potential weakening of its homogeneity advantage in the later stage. The Fe-200kDa-L10 group exhibited relatively high Γ’(*t*) in the early stage (≤7 d), indicating weaker initial inhibition. After 7 d, its Γ’(*t*) increase slowed markedly, with significantly enhanced inhibition, possibly due to the longer degradation cycle of high-molecular-weight PLA and its delayed action window. The hydroxyl- and carboxyl-terminated groups maintained relatively low Γ’(*t*) throughout the entire immersion period, with no significant difference between them.

### Mechanism-relevant studies of the effects of PLA coatings on iron corrosion via lowering local pH and inhibiting Ca-P layer

**Figure 7:**
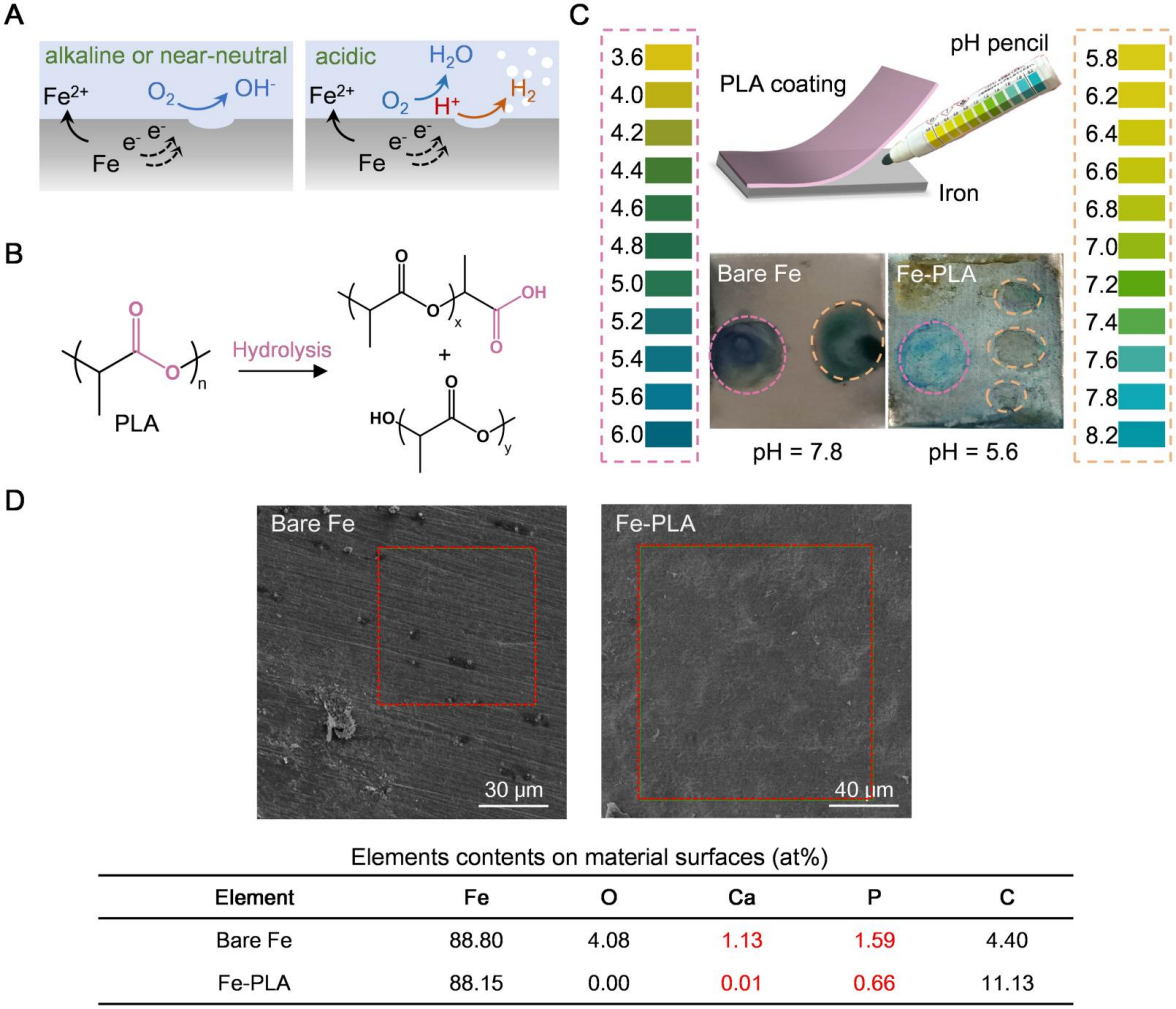
Mechanism of PLA coating in regulating iron corrosion. (**A**) Corrosion behavior of iron under varied pH conditions. (**B**) Degradation equation of PLA. (**C**) pH measurement on the surface of PLA-coated iron. (**D**) EDS elemental analysis of the PLA coating-iron interface, with analysis preferentially performed on uncorroded regions. The comparison indicates the existence of a Ca-P layer in Hank’s solution and its change after PLA coating.

After investigating the effects of PLA coatings with different parameters on the iron corrosion, we further analyzed the underlying mechanisms. As illustrated in Figure 7A, the corrosion of iron in Hank’s solution follows an electrochemical process. While anodic oxidation of iron occurs during corrosion, the cathodic reaction depends on the environmental pH. In alkaline or near-neutral environments, iron primarily undergoes oxygen absorption corrosion, characterized by the cathodic reduction of dissolved oxygen. In significant acidic environments, hydrogen evolution corrosion becomes dominant, accompanied by a minor cathodic process involving oxygen reduction to form water. The hydrolysis of PLA is illustrated in Figure 7B. The ester bonds in the polymer chain are cleaved by water, generating acidic degradation products, which may lower the local pH at the underlying metal surface. Consistent with this, our previous work has shown that the PLA coating on the iron substrate undergoes substantial degradation within a one-month immersion period, supporting the role of sustained polymer hydrolysis in modulating the local environment [36].

The local pH at the metal surface beneath the coating was measured, with results shown in Figure 7C. Using a pH pencil (range 5.8–8.2, orange dashed line), we found that the uncoated iron surface consistently exhibited a weakly alkaline pH ranging from 7.6–8.2. In contrast, the PLA-coated iron surface showed distinct acidity, with pH approaching the lower detection limit of the pencil. To obtain more accurate data, subsequent measurements were performed using pH pencil with a range of 3.6–6.0 (pink dashed line). The results indicated that the pH at the metal surface beneath the PLA coating was approximately 5.6. These findings illustrated that PLA generates a localized acidic microenvironment at the coating-metal interface through H^+^ release during its degradation, thereby accelerating the corrosion of iron. This acidic environment may promote the nucleation and propagation of corrosion pits, consequently increasing corrosion coverage.

To investigate the effect of PLA coating on the deposition of inorganic ions on the iron surface, EDS analysis was performed, with results shown in Figure 7D. After three days of immersion in Hank’s solution, the uncorroded regions of bare iron surface exhibited 1.13% Ca and 1.59% P, indicating the formation of a Ca-P deposition passivation layer. In contrast, the Ca and P contents in the uncorroded regions of PLA-coated iron surfaces were significantly lower, suggesting that the PLA coating effectively hindered the diffusion of Ca^2+^ and PO_4_^3−^ toward the iron substrate, thereby inhibiting the formation of the passivation layer. It is worthy of note that we had to use the casting method to prepare samples for interfacial pH measurements and EDS analysis because the PLA coating prepared by ultrasonic spraying remained firmly attached to the iron substrate and was thus difficult to peel off.

### Preparation parameters determine the switch time of PLA coatings from being protective to being corrosion-accelerating

**Figure 8:**
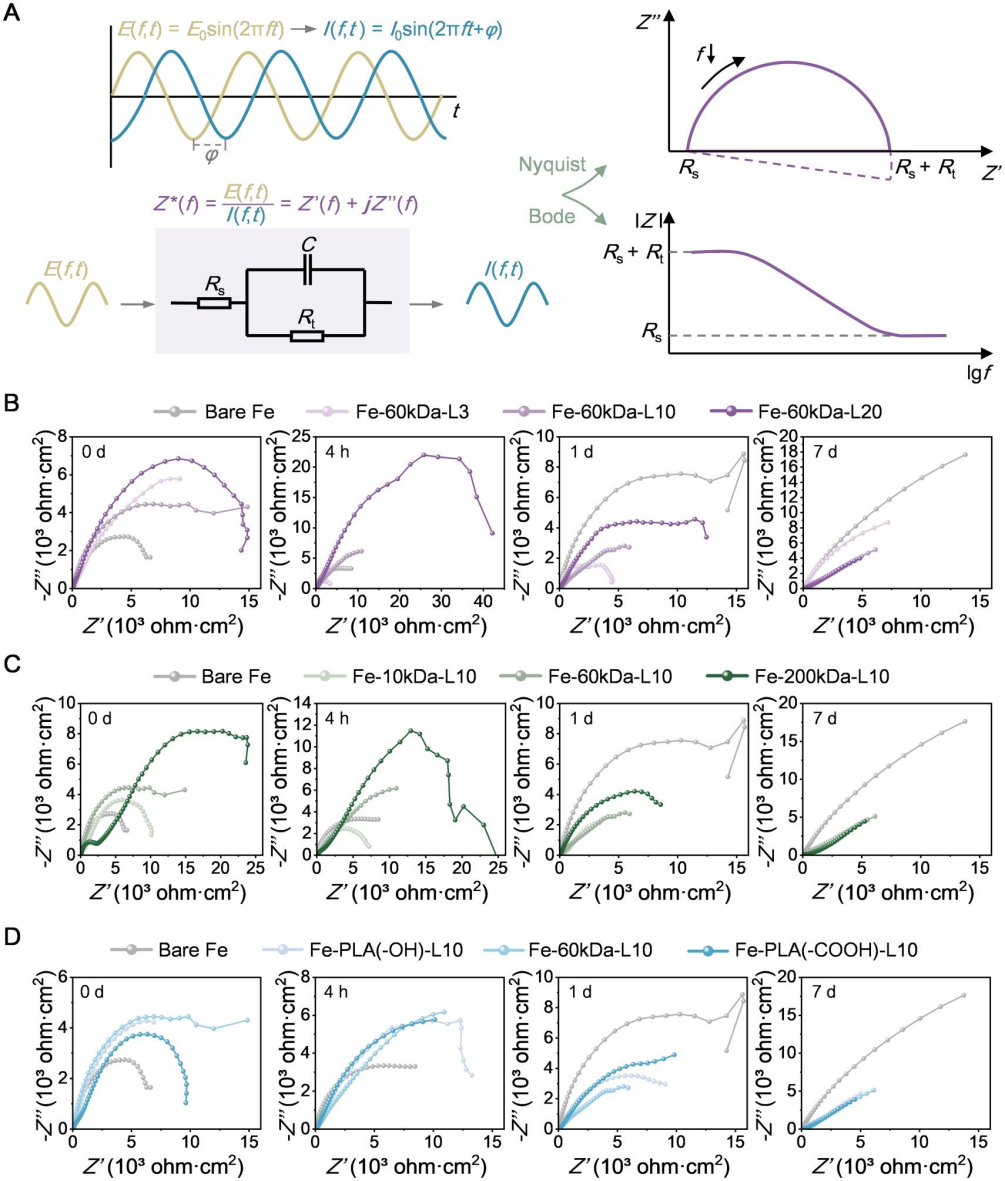
EIS results. (**A**) Schematic illustration of EIS measurement, the corresponding equivalent circuit model (comprising *R*_s_, *R*_t_ and *C*), and its representation in Nyquist and Bode plots. Here, *E* is the sinusoidal voltage, *f* is the frequency, *I* is the sinusoidal current density, *φ* is the phase shift between current and voltage, *R*_s_ is the solution resistance, *R*_t_ is the charge transfer resistance, *C* is constant phase element. *Z*’ and *Z*’’ denote the real and imaginary parts of the impedance (Z*), respectively. (**B–D**) Nyquist plots of PLA-coated iron with different (B) coating thickness, (C) molecular weight of PLA, (D) end group of PLA after varying immersion times in Hank’s solution.

Concurrently, we also observed that the PLA coating, acting as a physical barrier, exhibited a transient inhibitory effect on iron corrosion during the initial stage of immersion. To further investigate the influence of different PLA coating parameters on this initial inhibition behavior, EIS measurements were subsequently performed, with the principle illustrated in Figure 8A. The EIS results for all sample groups are presented as Nyquist plots (Figure 8B–D) and Bode plots (Supplementary Figures S9 and S10). Variations in the arc radii of the Nyquist plots indicate that all PLA-coated groups exhibited corrosion inhibition during the initial stage (0 d), with larger arc radii than that of the bare iron. Concurrently, the Bode plots revealed that the low-frequency impedance modulus of bare iron increased with immersion time, attributed to the gradual formation of a deposition passivation layer on its surface. In contrast, the low-frequency impedance of PLA-coated iron consistently decreased, indicating accelerated corrosion.

The thinnest Fe-60kDa-L3 group at 4 h of immersion exhibited an arc radius smaller than that of bare iron, marking its transition to corrosion acceleration, while the other two groups underwent this transition after 1 d. By 7 d, due to its lower PLA content and faster depletion, the accelerating effect of the Fe-60kDa-L3 group was weaker than that of thicker coating groups, with the Fe-60kDa-L20 group exhibiting the strongest acceleration and the lowest low-frequency impedance. Regarding molecular weight, the fastest-degrading Fe-10kDa-L10 group showed an arc radius smaller than that of bare iron at 4 h, transiting first to acceleration, followed by the Fe-60kDa-L10 and Fe-200kDa-L10 groups at 1 d. The accelerating effect of the Fe-200kDa-L10 group gradually strengthened with prolonged immersion. The arc radii of the three end group variants were similar at each time point. By 7 d of immersion, the carboxyl-terminated group exhibited slightly superior acceleration, reflected by the lowest low-frequency impedance.

The Bode plots based on phase angle (Supplementary Figure S10) showed that all PLA-coated iron samples exhibited phase angles below 30° in the high-frequency region during the initial immersion stage and maintained two-time-constant characteristics throughout the immersion period, indicating easy penetration of the electrolyte into the PLA coating and subsequent electrochemical reactions at the coating-iron substrate interface.

**Figure 9:**
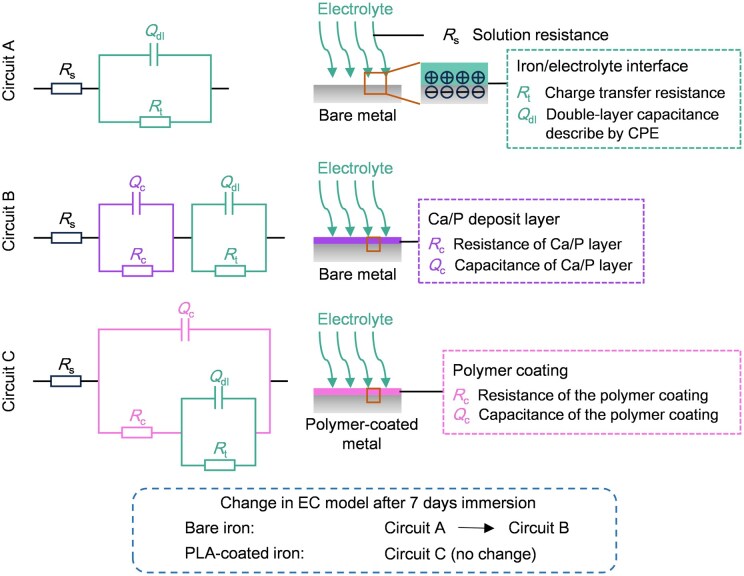
EC fitting models for bare iron electrodes and PLA-coated iron electrodes.

To further elucidate the corrosion process of the samples, EIS data were fitted using an equivalent circuit (EC), with the corresponding surface states of each electrode illustrated in Figure 9. In the EC parameters, *R*_s_ represents the solution resistance, *R*_t_ is the charge transfer resistance and *R*_c_ denotes the resistance of the deposition layer for bare iron or the polymer coating resistance for coated samples. Generally, larger *R*_t_ and *R*_c_ indicate higher corrosion impedance of the system, implying greater resistance to corrosion. Considering the inhomogeneity of the metal surface, a constant phase element (CPE) was introduced into the circuit, with *Q*_c_ and *Q*_dl_ representing the coating capacitance (or deposition layer capacitance) and the double-layer capacitance, respectively.

The obtained parameters are listed in Supplementary Table S1. At 0 d, all PLA-coated groups exhibited higher *R*_t_ than bare iron, confirming the transient inhibition effect of the coatings in the initial stage. Regarding coating thickness, the Fe-60kDa-L3 group, with lower PLA content, showed a gradual increase in *R*_c_ and *R*_t_ in the later stage, indicating a weakening of corrosion promotion over time. In contrast, the *R*_t_ of the Fe-60kDa-L20 group continuously decreased, reaching the lowest value among the thickness groups at 7 d, demonstrating the most significant acceleration effect in the later stage. Regarding molecular weight, the Fe-10kDa-L10 group maintained the lowest *R*_t_ throughout the immersion period, approximately one order of magnitude lower than other groups, exhibiting the strongest corrosion promotion. The *R*_t_ of the Fe-200kDa-L10 group decreased markedly in the later stage, suggesting potential for enhanced long-term acceleration. Regarding end group, the *R*_t_ trends of the three groups were generally similar, all decreasing gradually over time. By 7 d, the carboxyl-terminated group showed a slightly lower *R*_t_ than the other two groups, indicating a marginally superior acceleration effect.

## Discussion

Significant progress has been made in medical devices with the development of biomaterials [51–55]. Surface modification has been applied to tune cell-material interactions [56–61]. The present study distinguishes itself in technology to employ polymer coatings on metal surfaces to adjust corrosion in a biomimetic medium, motivated by the development of iron-based biodegradable stents, which has long been constrained by excessively slow corrosion rate and unpredictable corrosion behavior [62–64]. We found that the PLA coating can not only increase the corrosion rate of iron by approximately three folds but also reduce corrosion inhomogeneity by more than three orders of magnitude, with coating parameters significantly influencing the regulatory effects.

**Figure 10:**
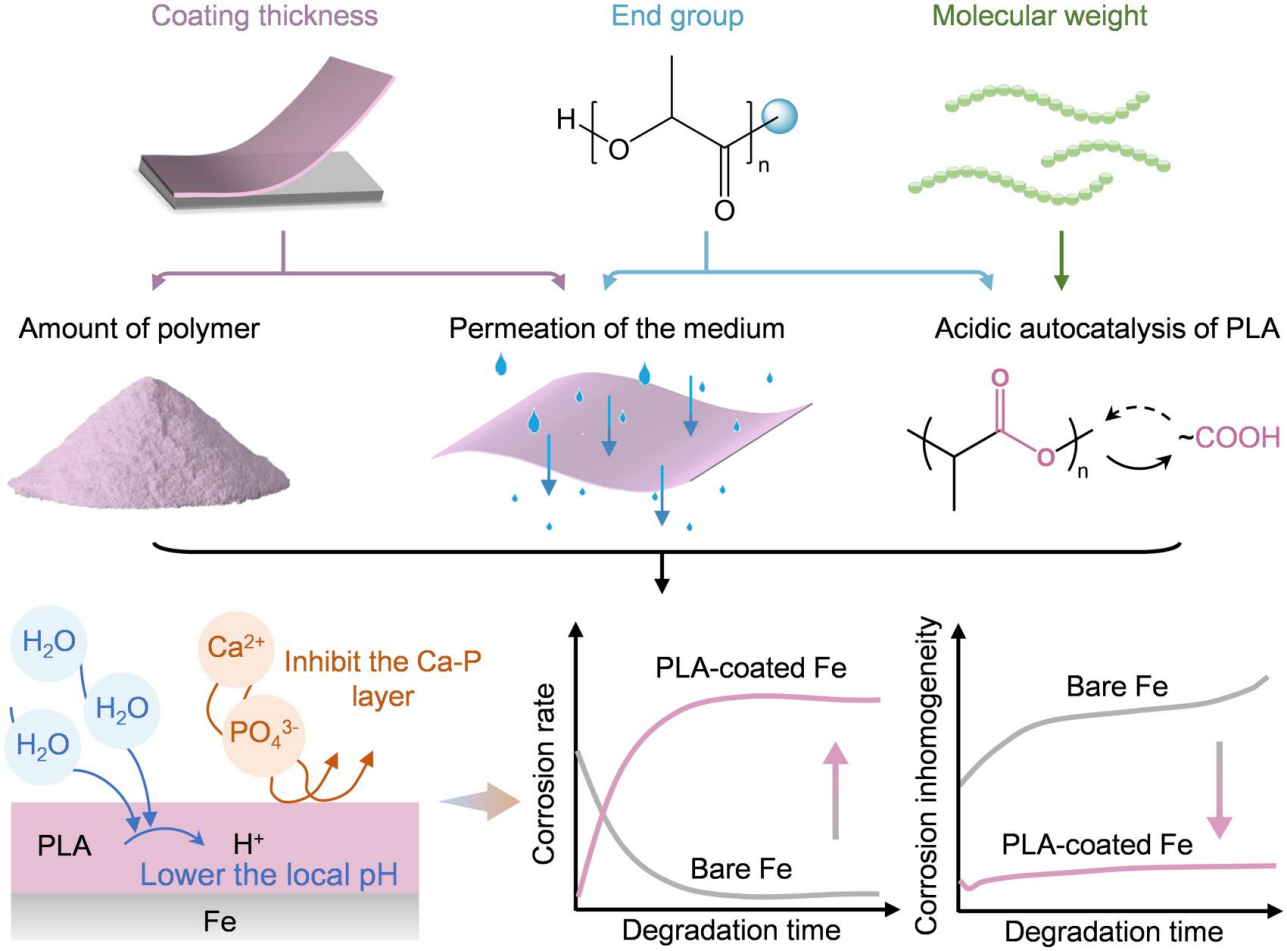
Schematic illustration depicting the influence of distinct coating parameters on the corrosion modulation of iron.

Coating thickness determines the integrity of the film and the total amount of PLA (Figure 10). The former affects the permeation of the immersion medium, and the latter influences the total amount of acid produced by hydrolysis (i.e. local pH). Therefore, a thicker coating initially hinders the ingress of the immersion medium, resulting in slower initial corrosion. This is corroborated by the lower initial *i*_corro_ observed in the Fe-60kDa-L20 group. In the later stage, however, the thick coating impedes the protective barrier effect of the Ca-P layer and generates a substantial amount of acidic byproducts, leading to a drop in local pH, which significantly modulates iron corrosion. The Fe-60kDa-L20 group exhibited the highest corrosion rate after 30 days of immersion, with its Γ reduced by more than 9000 times compared to that of pure iron, alongside the lowest Γ’ value.

The end groups of the polymer influence the polarity of the coating, thereby affecting its wettability by the immersion medium and, in some cases, the pH (Figure 10). When the end groups are highly polar, the immersion medium readily permeates the film, promoting corrosion. When the end groups themselves are carboxyl groups, the initial pH is lowered, further facilitating corrosion. Consequently, ester-terminated PLA, being uncharged and exhibiting low polarity, results in the slowest corrosion. Hydroxyl-terminated polymer does not alter the pH but generally possess higher polarity than ester-terminated one, leading to faster corrosion of the underlying metal. Carboxyl-terminated polymer, with a significant increase in polarity and a direct reduction in pH, is expected to yield the fastest corrosion. The Fe-PLA(-COOH)-L10 group exhibited the lowest *R*_t_ and the best homogeneity in the later stage. Nevertheless, end groups constitute only a small fraction of the entire polymer. Given the relatively high molecular weight of the polymers used in this work, the effects of end groups may not be pronounced.

The influence of molecular weight can be mainly explained by the difference in end-group concentration. With a certain mass, a lower molecular weight corresponds to a greater number of polymer chains and thus a higher total end-group concentration. In this study, the end-group concentration of PLA with MW 10 kDa was 20 times that of 200 kDa, which can significantly affect the hydrolysis rate. More critically, during degradation, each hydrolysis of an ester bond in PLA generates one hydroxyl end group and one carboxyl end group (Figure 7B). As hydrolysis proceeds, the end-group composition of the polymer evolves, with carboxyl end groups gradually accumulating. Since the hydrolysis of PLA is an acidic autocatalytic process [65], once carboxyl end groups are generated with hydrolysis of each ester bond, the hydrolysis rate accelerates (Figure 10). Consequently, the modulatory effect of low-molecular-weight PLA on iron corrosion is amplified. The Fe-10kDa-L10 group thus exhibited the highest *i*_corro_ throughout the immersion period, the fastest growth in corrosion coverage, and the lowest Γ, indicating the most uniform corrosion process.

PLA coatings exhibited a time-dependent effect on iron corrosion. In the early stage (0–4 h), physical barrier effects inhibited corrosion. With prolonged immersion, coating hydrolysis led to a decreased interfacial pH and inhibition of Ca-P deposition layer formation, accelerating corrosion. The transition time was modulated by coating parameters. Thin coatings and low-molecular-weight groups transitioned earlier, while thick coatings and high-molecular-weight groups exhibited longer inhibition periods with progressively stronger later-stage promotion.

Quantification of corrosion inhomogeneity using the static parameter Γ and dynamic parameter Γ’(*t*) revealed that PLA coatings reduced Γ by more than three orders of magnitude, with Γ’(*t*) consistently maintained at ≤1 μm, indicating significant suppression of local longitudinal pit development. Moreover, in investigating the evolution trend of Γ’(*t*), we observed that PLA-coated groups typically exhibit a ‘first decrease then increase’ U-shaped characteristic during the initial corrosion stage. To explain this phenomenon, we expand the expression of Γ’(*t*) using equations (1) and (2) as follows:


(7) Γ′(t)=dcorro(t)2θ(t)=atb2(1-e-k'tn')

As *t* → 0, Γ’(*t*) can be expressed as:


(8) Γ'(t)=a2k'tb-n′

As θ → 100%, Γ’(*t*) can be expressed as:


(9) Γ'(t)=dcorro(t)2=atb2

Therefore, Γ’(*t*) reflects well the extent of corrosion inhomogeneity only if coverage is not close to 100%.

**Figure 11:**
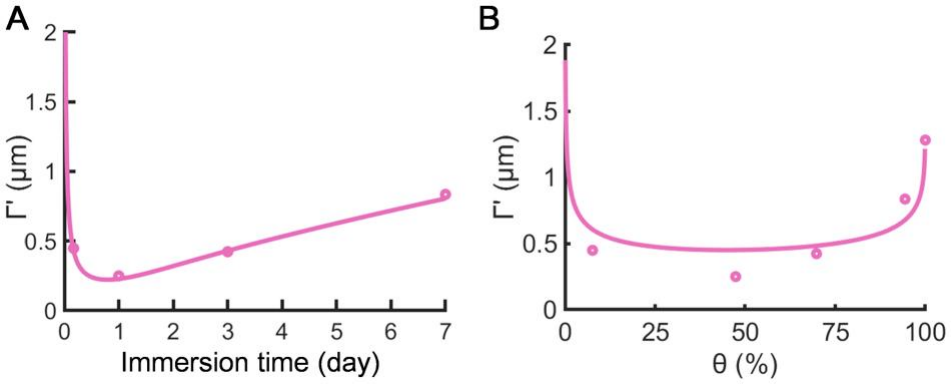
Temporal evolution of instantaneous corrosion inhomogeneity for Fe-10 kDa-L10 after immersion in Hank’s solution. (**A**) Γ’(*t*) curve. (**B**) Γ’(θ) curve.

Taking the Fe-10kDa-L10 group as an example, the Γ’(*t*) curve exhibits a typical U-shaped characteristic (Figure 11A). During the initial immersion period, the PLA coating induces the formation of numerous corrosion pits on the iron surface. At this stage, the corrosion is dominated by the rapid increase in θ, while the growth rate of *d*_corro_ lags behind, resulting in a brief decrease in Γ’. As the immersion time extends, θ gradually approaches saturation, and the corrosion transitions to being dominated by the deepening of existing corrosion pits. Consequently, *d*_corro_ continues to increase, and Γ’ enters a sustained rising phase.

We further establish the relation between Γ’ and θ after combining equations (4) and (6), resulting in:


(10) Γ'(θ)=12θ[-ln⁡(1-θ)k]1n

This expression directly relates the dynamic inhomogeneity to the corrosion coverage, circumventing the influence of the time parameter and facilitating analysis of inhomogeneity evolution from the perspective of ‘corrosion progression’. Using Fe-10kDa-L10 as an example, Figure 11B presents the Γ’(θ) curve with a U-shaped characteristic again. This result demonstrates that 50% coverage lies in a relatively stable region of Γ’ versus θ, which rationalizes our suggestion to take corrosion depth at θ = 50% as the default measure of corrosion inhomogeneity as expressed in equation (5).

This study establishes a quantitative correlation between PLA coating parameters and iron corrosion, providing a foundation for optimizing the degradation of iron-based MPS. Nevertheless, *in vitro* biomimetic conditions cannot fully replicate the complex *in vivo* environment, and the 30-day observation period provides limited coverage of the complete stent degradation process. Subsequent studies incorporating animal models and extended time scales are warranted. Furthermore, although the PLA coating prepared by ultrasonic spraying in this study exhibited sufficient adhesion stability, systematic evaluation of the coating spray process and interfacial adhesion strength is still required during future product development and clinical translation to further ensure its reliability.

## Conclusion

In this study, we systematically investigated the effects of coating thickness, molecular weight, and end group of PLA on iron corrosion under biomimetic *in vitro* conditions. After proposing dynamic corrosion inhomogeneity, the extended equation set including eight equations (equations 1–7, 10) has been summarized to describe random degradation, and employed to quantitatively compare the corrosion rate and inhomogeneity of iron under different parameters of PLA coatings. The results demonstrate that, through the dual mechanisms of interfacial acidification and inhibition of Ca-P passivation layer formation, PLA coatings can increase the corrosion rate of iron by approximately three folds whereas reducing corrosion inhomogeneity by more than three orders of magnitude. Coating thickness primarily governs the physical barrier effect and the total amount of PLA, with the former affecting medium permeation and the latter determining the total acid production via hydrolysis, thereby influencing the intensity and duration of corrosion regulation. The molecular weight and end group of PLA collectively dominate its hydrolysis rate, and the effect of molecular weight is essentially attributed to the concentration of end groups and the autocatalytic nature of hydrolysis. End group types also affect the polarity of the coating, thereby influencing medium permeation. Future studies may further explore the synergistic regulation of the ‘quantity’ and ‘rate’ of PLA coatings to guide the clinical application of iron-based MPS. The new insight and methodology might be helpful for development and research of other degradable materials applied in medical devices, drug delivery systems, ships and beyond.

## Supplementary Material

rbag126_Supplementary_Data
