# Supplementary material for: Effects of molecular weight, end group of polylactide and coating thickness on corrosion rate and inhomogeneity of iron under biomimetic conditions
Source: Regen Biomater. 2026 Jun 13;13:rbag126. doi: 10.1093/rb/rbag126 (PMC13348712; doi:10.1093/rb/rbag126)
Supplement: rbag126_Supplementary_Data [file rbag126_supplementary_data.pdf]

Supplementary Materials for

Effects of molecular weight, end group of polylactide and coating thickness on corrosion rate and inhomogeneity of iron under biomimetic condition

Wenjie Wu, Jiandong Ding*

State Key Laboratory of Molecular Engineering of Polymers, Department of Macromolecular Science, Fudan University, Shanghai 200438, China

* Correspondence address. E-mail: jdding1@fudan.edu.cn (J.D. Ding)

This file includes

Figures S1 to S10

Table S1

Supplementary results

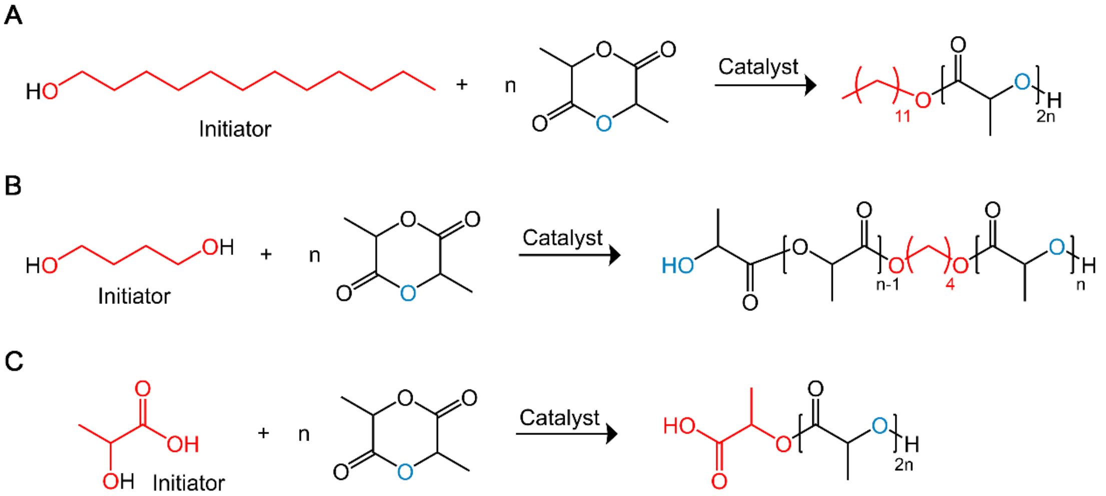

Figure S1. Synthetic routes for three types of PLA with (A) ester, (B) hydroxyl, and (C) carboxyl terminal groups. The distinct terminal groups arise from the different initiators. In this study, D,L lactide were fed prior to ring opening polymerization.

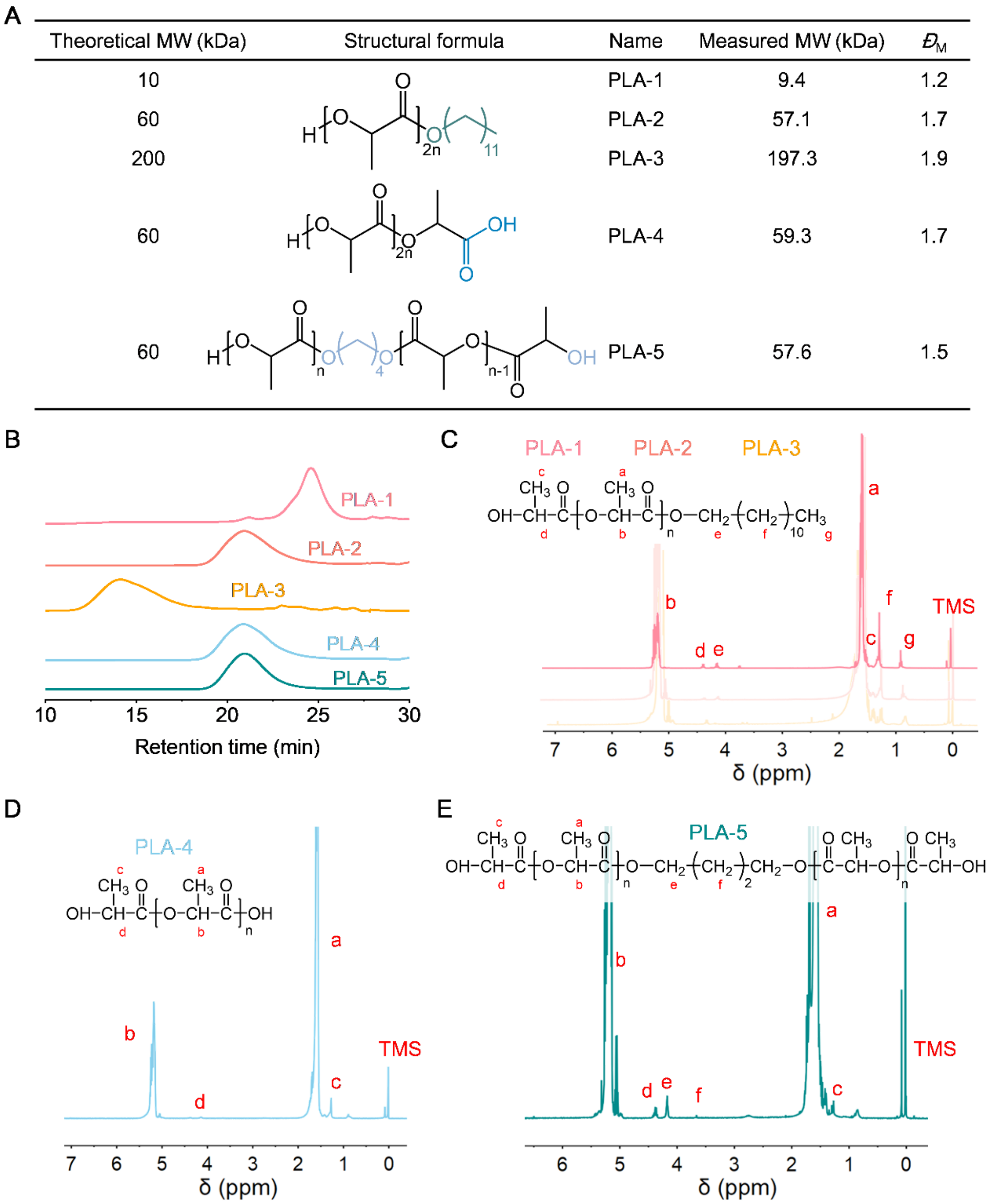

Figure S2. Molecular weights and structural characterization of different PLA samples. (A) Chemical structures of PLA with different end groups, along with their experimental molecular weights and molar mass dispersity ($Đ_M$) reflecting molecular weight distribution. For convenience, they are denoted as PLA 1-5. (B) GPC results of different PLA samples. (C-E) NMR spectra of PLA with (C) ester end-groups, (D) carboxyl end-group and (E) hydroxyl end-group. Characteristic peaks corresponding to distinct proton types are labeled in the spectra.

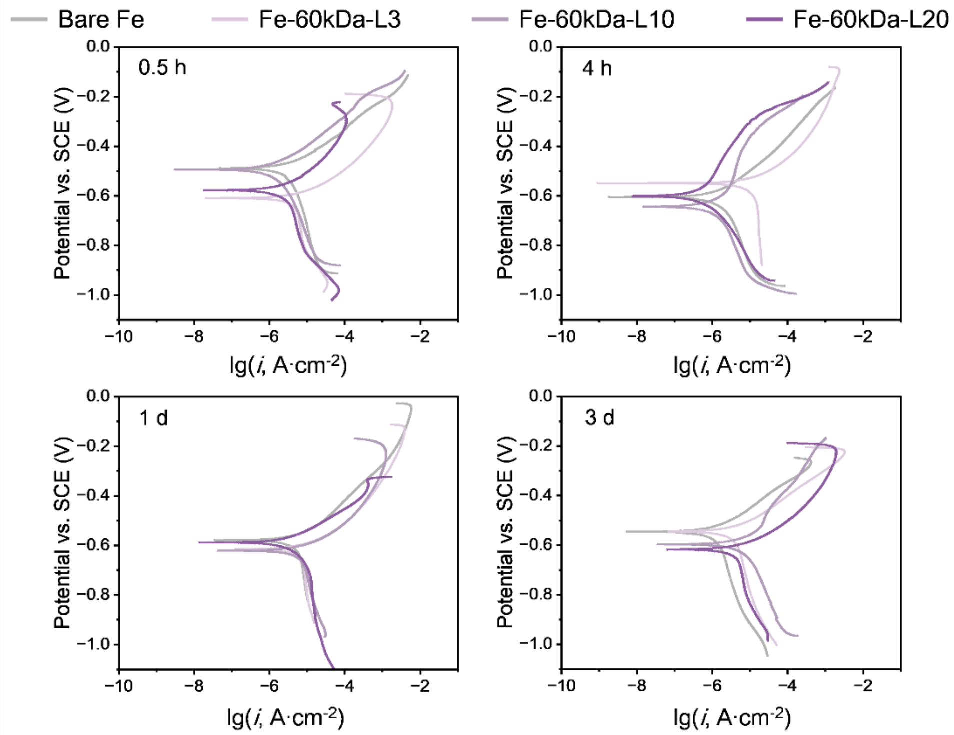

Figure S3. Tafel curves of PLA-coated iron with different coating thicknesses after immersion in Hank's solution for 0.5 h, 4 h, 1 d and 3 d.

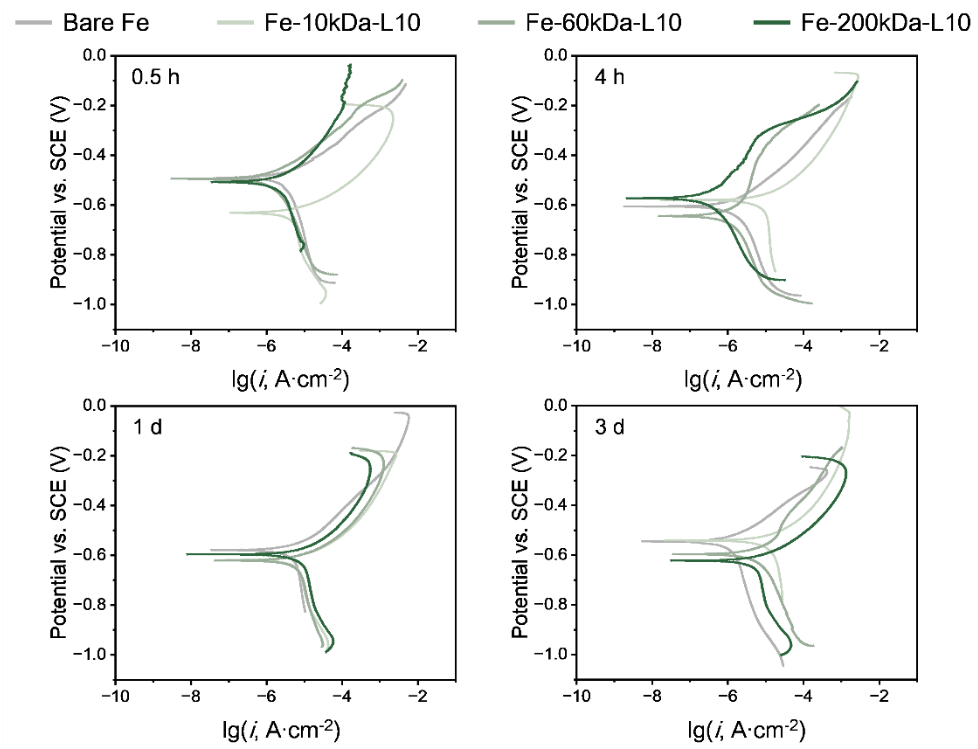

Figure S4. Tafel curves of PLA-coated iron with different PLA molecular weight after immersion in Hank's solution for 0.5 h, 4 h, 1 d and 3 d.

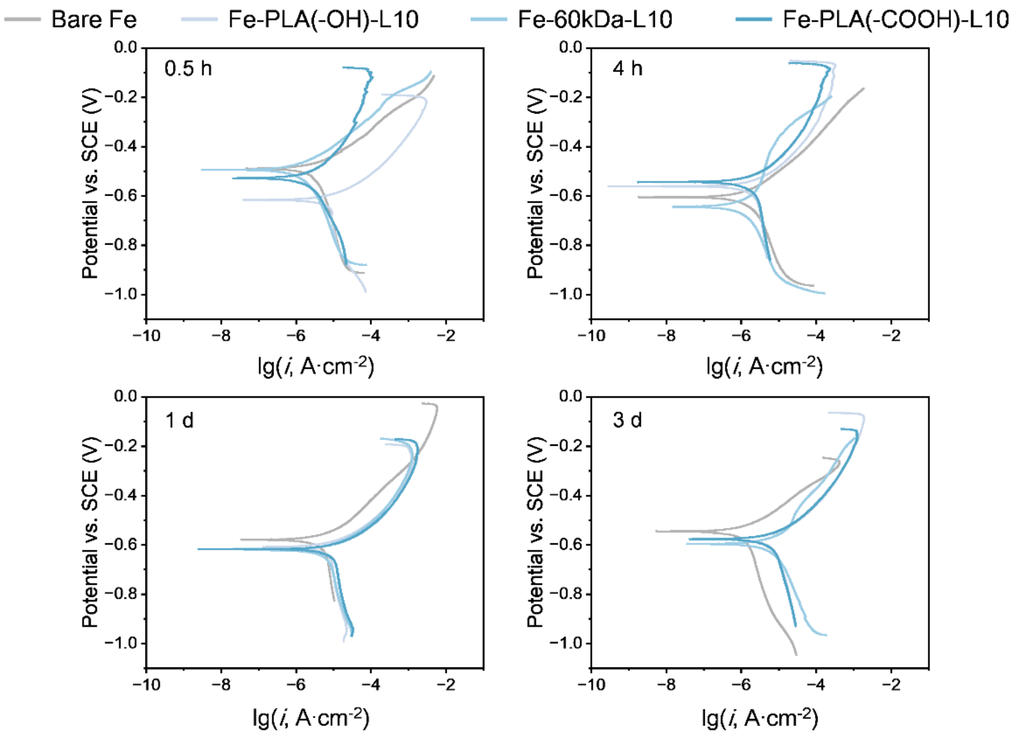

Figure S5. Tafel curves of PLA-coated iron with different PLA end group after immersion in Hank's solution for 0.5 h, 4 h, 1 d and 3 d.

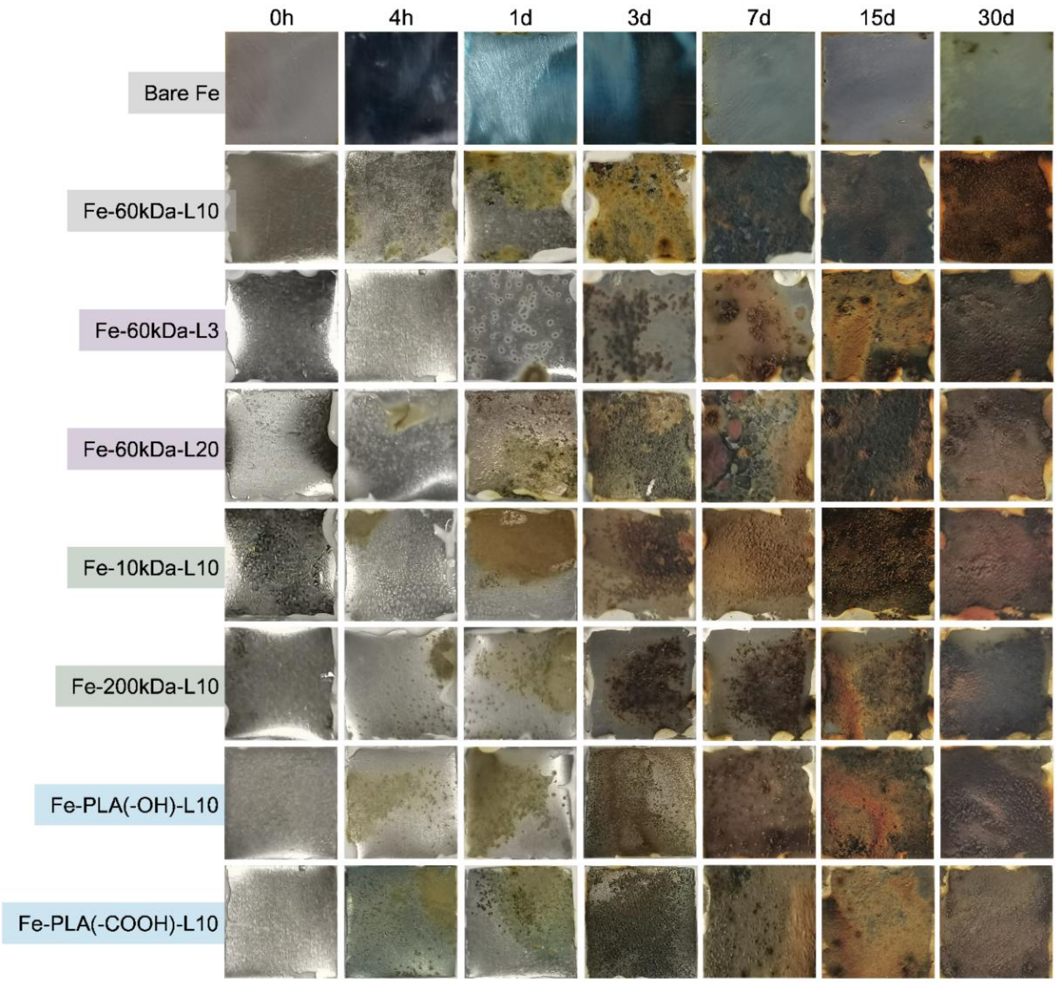

Figure S6. Optical images showing surface corrosion coverage on PLA-coated iron sheets with varying parameters after immersion in Hank's solution for different times.

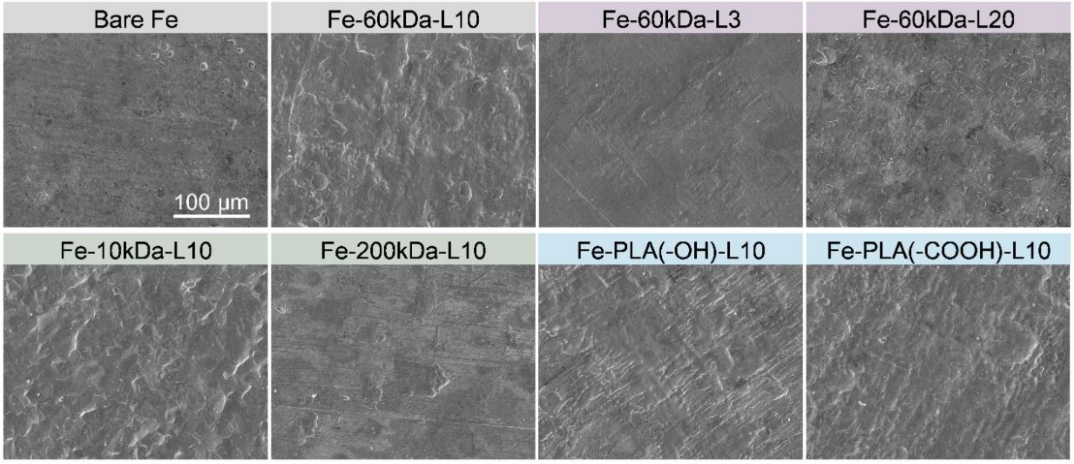

Figure S7. Surface SEM images of iron sheets with varied PLA coatings after 15 d immersion in Hank's solution.

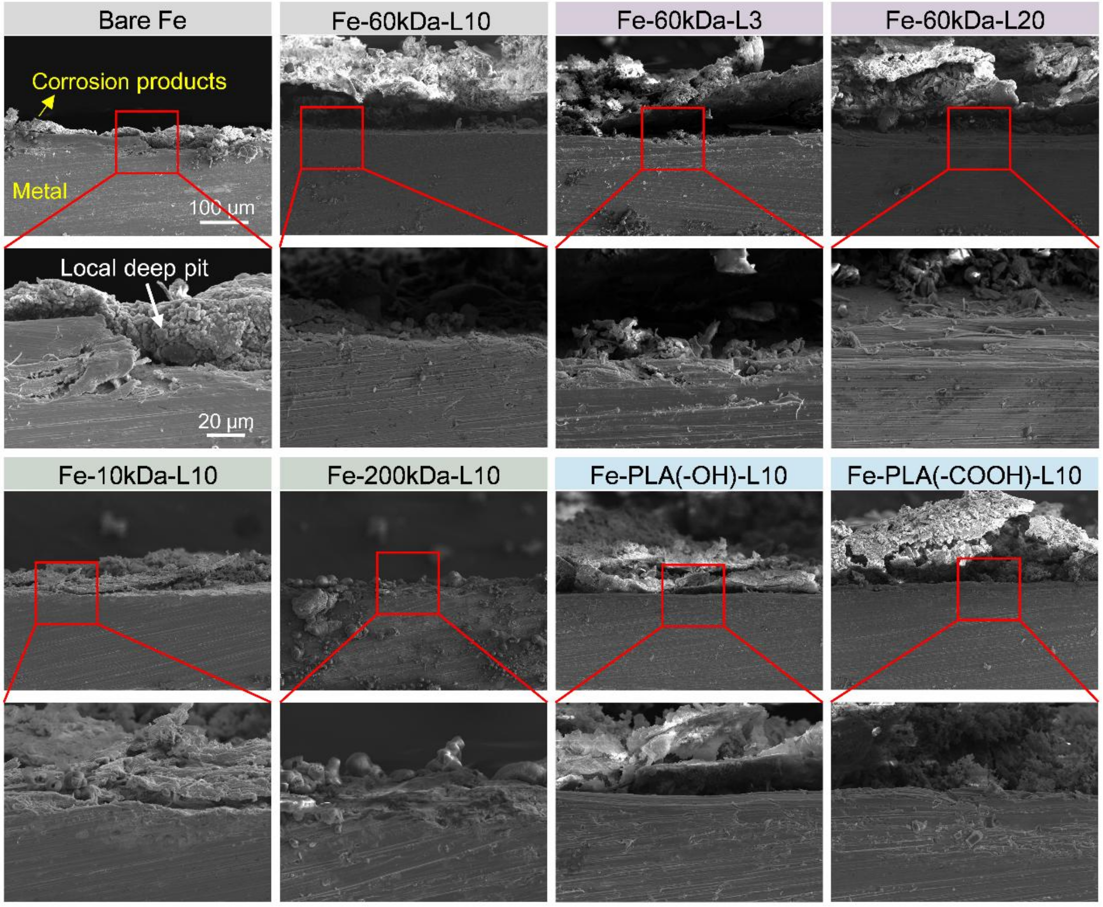

Figure S8. Cross-sectional SEM images of iron sheets with varied PLA coatings after 15 d immersion in Hank's solution. An obvious localized corrosion pit was observed on the cross-section of bare iron and indicated by an arrow, whereas all PLA-coated iron samples exhibited a relatively continuous corrosion front in cross-sectional views.

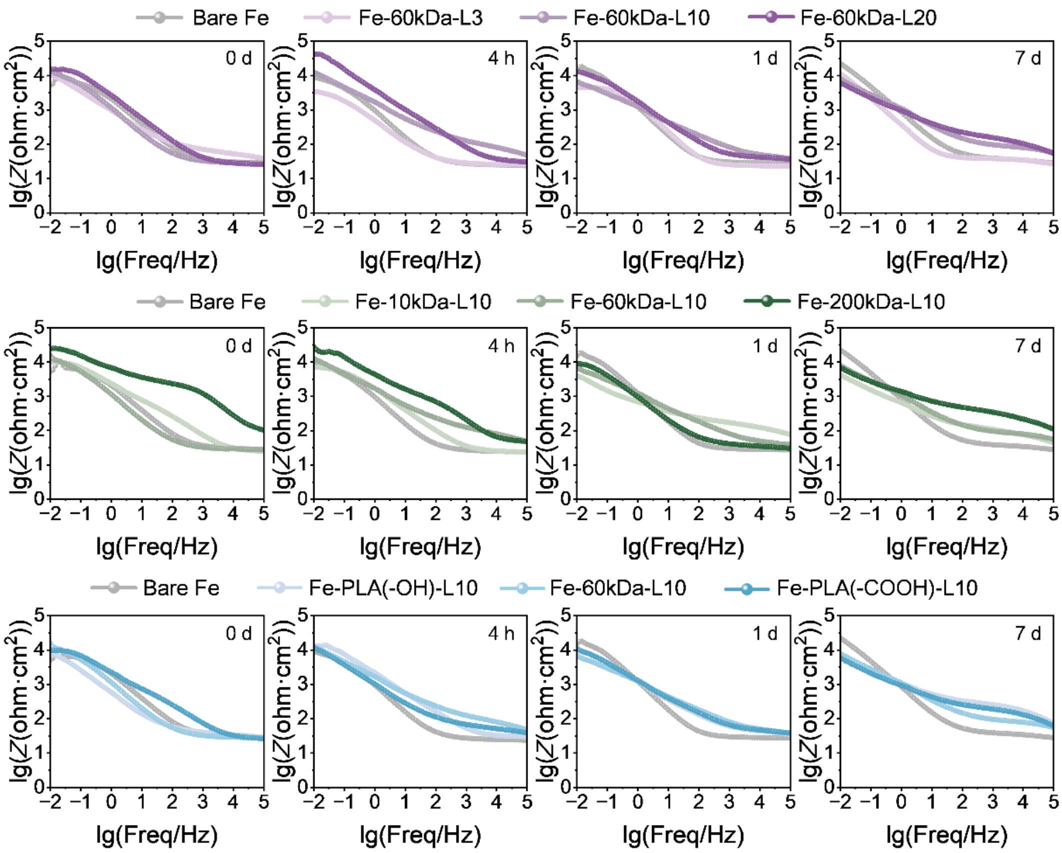

Figure S9. Bode plots of iron electrodes coated with PLA of different parameters before immersion and after 4 h, 1 d, and 7 d of immersion in Hank's solution, with the impedance magnitude |*Z*| plotted on the y-axis. From top to bottom, the plots correspond to PLA coatings with different thicknesses, molecular weights, and end groups, respectively.

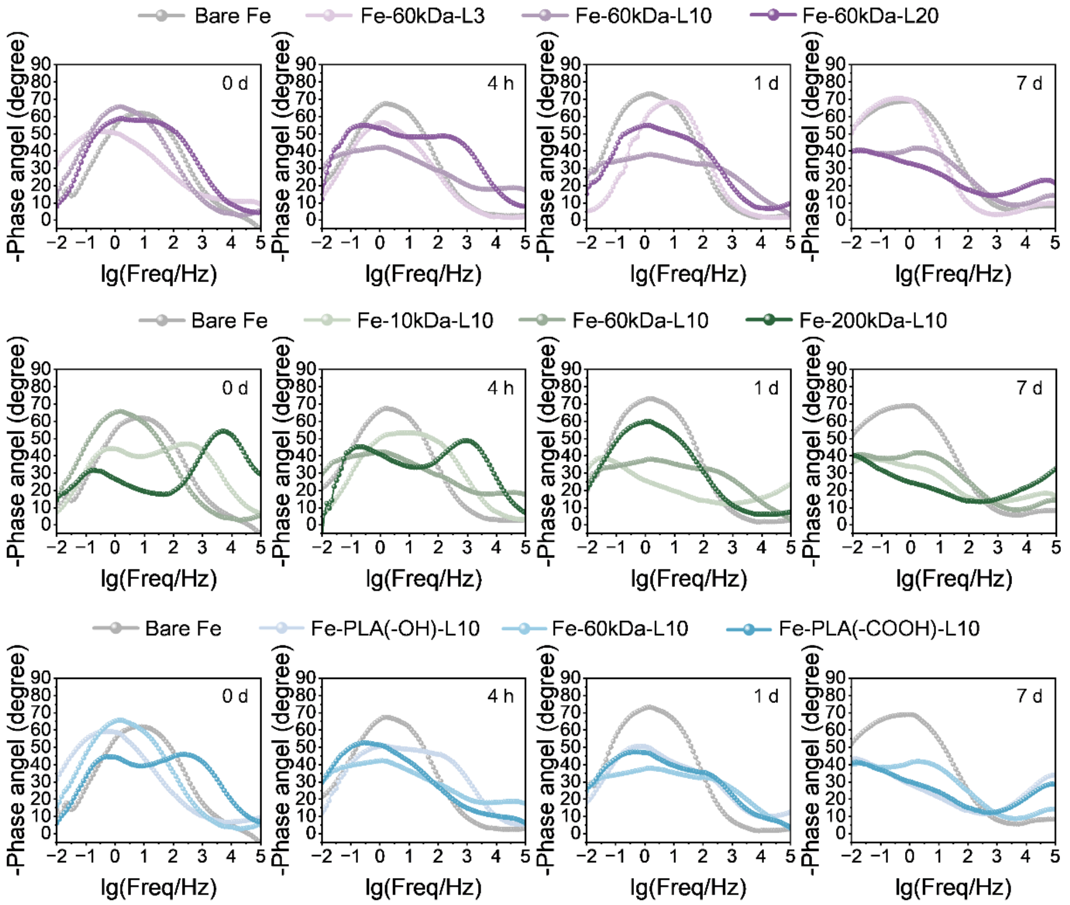

Figure S10. Bode plots of iron electrodes coated with PLA of different parameters before immersion and after 4 h, 1 d, and 7 d of immersion in Hank's solution, with the phase angle φ plotted on the y-axis. From top to bottom, the plots correspond to PLA coatings with different thicknesses, molecular weights, and end groups, respectively.

Table S1. The fitted parameters of equivalent circuits for each group.

		Y_{0c} ($S·cm^{-2}·s^n$)	n_c	R_c ($\Omega·cm^2$)	Y_{0dl} ($S·cm^{-2}·s^n$)	n_{dl}	R_t ($\Omega·cm^2$)	χ^2
Bare Fe	Day 0 (Circuit A)	-	-	-	1.2×10^{-4}	0.75	8.6×10^{3}	8.0×10^{-3}
	Day 3 (Circuit B)	8.2×10^{-5}	0.64	1.7×10^{1}	8.9×10^{-5}	0.91	3.0×10^{4}	1.9×10^{-3}
	Day 7 (Circuit B)	9.1×10^{-4}	0.21	4.8×10^{1}	2.9×10^{-4}	0.79	5.4×10^{4}	9.1×10^{-4}
Fe-60kDa-L10	Day 0 (Circuit C)	1.4×10^{-4}	0.77	6.0×10^{1}	6.6×10^{-5}	0.79	1.3×10^{4}	7.4×10^{-4}
	Day 3 (Circuit C)	3.7×10^{-8}	1.00	3.7×10^{1}	3.4×10^{-4}	0.36	1.3×10^{4}	2.5×10^{-3}
	Day 7 (Circuit C)	1.8×10^{-6}	0.75	4.4×10^{1}	3.2×10^{-4}	0.57	6.8×10^{3}	4.0×10^{-4}
Fe-60kDa-L3	Day 0 (Circuit C)	4.8×10^{-5}	0.63	5.6×10^{1}	2.6×10^{-4}	0.66	1.2×10^{4}	1.2×10^{-3}
	Day 3 (Circuit C)	2.0×10^{-4}	0.62	0.7×10^{1}	7.9×10^{-4}	0.81	1.6×10^{4}	1.0×10^{-3}
	Day 7 (Circuit C)	9.3×10^{-5}	0.46	2.3×10^{1}	5.6×10^{-4}	0.85	4.3×10^{4}	4.3×10^{-4}
Fe-60kDa-L20	Day 0 (Circuit C)	8.3×10^{-5}	0.72	3.9×10^{3}	1.4×10^{-5}	0.93	1.5×10^{4}	1.7×10^{-3}
	Day 3 (Circuit C)	4.4×10^{-5}	0.69	6.1×10^{1}	1.8×10^{-4}	0.72	1.5×10^{4}	1.1×10^{-3}
	Day 7 (Circuit C)	1.5×10^{-5}	0.56	1.4×10^{2}	3.8×10^{-4}	0.52	2.6×10^{3}	3.1×10^{-4}
Fe-10kDa-L10	Day 0 (Circuit C)	4.4×10^{-5}	0.68	1.1×10^{3}	9.1×10^{-5}	0.70	1.1×10^{4}	6.3×10^{-4}
	Day 3 (Circuit C)	3.0×10^{-4}	0.73	1.7×10^{2}	8.9×10^{-5}	0.73	4.8×10^{3}	4.7×10^{-4}
	Day 7 (Circuit C)	2.5×10^{-4}	0.34	1.7×10^{2}	2.3×10^{-4}	0.70	9.5×10^{2}	4.2×10^{-4}
Fe-200kDa-L10	Day 0 (Circuit C)	6.9×10^{-7}	0.78	2.3×10^{3}	7.2×10^{-5}	0.52	3.7×10^{4}	1.5×10^{-3}
	Day 3 (Circuit C)	7.4×10^{-5}	0.45	1.7×10^{2}	2.3×10^{-4}	0.54	3.5×10^{4}	2.4×10^{-3}
	Day 7 (Circuit C)	2.0×10^{-5}	0.44	4.4×10^{2}	3.9×10^{-4}	0.43	6.6×10^{3}	1.0×10^{-3}
Fe-PLA(-OH)-L10	Day 0 (Circuit C)	1.2×10^{-5}	0.63	1.9×10^{1}	4.3×10^{-4}	0.70	1.6×10^{4}	1.8×10^{-3}
	Day 3 (Circuit C)	3.4×10^{-5}	0.77	1.3×10^{3}	1.2×10^{-4}	0.61	1.3×10^{4}	1.4×10^{-3}
	Day 7 (Circuit C)	5.3×10^{-6}	0.58	2.6×10^{2}	4.6×10^{-4}	0.47	5.1×10^{3}	8.5×10^{-4}
Fe-PLA(-COOH)-L10	Day 0 (Circuit C)	5.1×10^{-5}	0.66	1.2×10^{3}	8.9×10^{-5}	0.74	1.1×10^{4}	9.2×10^{-4}
	Day 3 (Circuit C)	7.6×10^{-5}	0.66	2.4×10^{2}	1.5×10^{-4}	0.50	1.3×10^{4}	1.3×10^{-3}
	Day 7 (Circuit C)	5.0×10^{-6}	0.62	1.7×10^{2}	5.1×10^{-4}	0.46	5.0×10^{3}	6.0×10^{-4}
